# Changes in the metabolic potential of the sponge microbiome under ocean acidification

**DOI:** 10.1038/s41467-019-12156-y

**Published:** 2019-09-12

**Authors:** Emmanuelle S. Botté, Shaun Nielsen, Muhammad Azmi Abdul Wahab, John Webster, Steven Robbins, Torsten Thomas, Nicole S. Webster

**Affiliations:** 1 Australian Institute of Marine Science, Townsville, QLD 4810 Australia; 2 Centre for Marine Science and Innovation, School of Biological, Earth and Environmental Sciences, University of New South Wales, Sydney, NSW 2052 Australia; 3 Australian Centre for Ecogenomics, University of Queensland, St Lucia, QLD 4072 Australia; 4 Present Address: Australian Institute of Marine Science, Arafura Timor Research Facility, Brinkin, NT 0810 Australia

**Keywords:** Metagenomics, Water microbiology, Microbial ecology, Marine biology

## Abstract

Anthropogenic CO_2_ emissions are causing ocean acidification, which can affect the physiology of marine organisms. Here we assess the possible effects of ocean acidification on the metabolic potential of sponge symbionts, inferred by metagenomic analyses of the microbiomes of two sponge species sampled at a shallow volcanic CO_2_ seep and a nearby control reef. When comparing microbial functions between the seep and control sites, the microbiome of the sponge *Stylissa flabelliformis* (which is more abundant at the control site) exhibits at the seep reduced potential for uptake of exogenous carbohydrates and amino acids, and for degradation of host-derived creatine, creatinine and taurine. The microbiome of *Coelocarteria singaporensis* (which is more abundant at the seep) exhibits reduced potential for carbohydrate import at the seep, but greater capacity for archaeal carbon fixation via the 3-hydroxypropionate/4-hydroxybutyrate pathway, as well as archaeal and bacterial urea production and ammonia assimilation from arginine and creatine catabolism. Together these metabolic features might contribute to enhanced tolerance of the sponge symbionts, and possibly their host, to ocean acidification.

## Abstract (web summary)

Anthropogenic CO_2_ emissions are causing ocean acidification, which can affect the physiology of some organisms. Here, Botté et al. use metagenomics to show differences in metabolic potential between sponge microbiomes sampled at a shallow volcanic CO_2_ seep and those from nearby control sites.

## Introduction

Nearly one-third of anthropogenic CO_2_ has been taken up by the oceans^1^, resulting in lower concentrations of carbonate ions and higher concentrations of bicarbonate ions (HCO_3_^−^) and protons (H^+^). Such changes to ocean chemistry have led to a 0.1 unit reduction in seawater pH since pre-industrial times^2^, a phenomenon known as ocean acidification (OA). As atmospheric CO_2_ levels continue to rise, oceans are projected to acidify further^2^, with significant consequences expected for sensitive ecosystems such as coral reefs, which depend on carbonate ions for future reef accretion^3^. However, changes in biological and ecological processes resulting from OA can be difficult to predict, partly because the vast majority of studies have examined the biological and physiological effects of OA using short-term experimental exposures. An alternative approach is to investigate long-term effects of projected OA using volcanic CO_2_ seeps which feature naturally reduced seawater pH^4–6^. Research conducted at natural CO_2_ seeps over the past decade has revealed substantial changes in the population structure of a wide range of organisms^4,5^. Studies have assessed impacts on species diversity and abundance^4,5,7,8^, gene expression^9^, as well as functional potential of eukaryotic communities^10^. From a microbial perspective, OA has also been shown to modify the composition of the microbiome in some reef species^11–13^.

Microbes play vital roles in the health and survival of all marine organisms^14,15^. One of the best understood reef microbial symbioses is that which occurs between sponges and their associated bacteria and archaea. Sponges often host abundant, diverse and species-specific microbial communities^16^, thought to undertake essential roles in nutrient cycling, vitamin production and natural product biosynthesis^17,18^. Shifts in microbial communities have been detected in sponges collected from a natural CO_2_ seep at Upa-Upasina within the Milne Bay Province, Papua New Guinea (PNG). At this location, the shallow reef comprises a control site with “ambient” pH (median 8.01) representing a healthy and diverse reef system, and a seep site located 500 m away, featuring “reduced” pH (median 7.77) caused by the release of > 99% pure CO_2_ gas, which has resulted in significantly reduced species diversity and habitat complexity^5,19^. Two sponge species exhibit drastically different distribution and abundance patterns at Upa-Upasina. The seep-tolerant *Coelocarteria singaporensis* is 40-times more abundant at the seep, whereas the seep-sensitive *Stylissa flabelliformis* is 6-times more abundant at the control site^5^. Microbial community analysis revealed variation in the microbiome of *C. singaporensis* between sites, whereas the microbial communities of *S. flabelliformis* were largely conserved across both environments^13^.

Understanding how the sponge microbiome responds to long-term environmental change has been recognised as critical for anticipating the fate of sponge symbioses under predicted climate scenarios^20,21^. Yet, whilst changes in microbial community composition have been found under OA, the impact on the functional potential of symbiont communities is currently unknown.

To address this question, we examined the microbiomes of *C. singaporensis* and *S. flabelliformis* at Upa-Upasina. We specifically investigated whether the functional potential of the symbiotic microbiomes varied between control and seep sites and if these changes could contribute to maintaining a successful symbiosis in acidified oceans.

Here we find that the microbiome of the seep-tolerant *C. singaporensis* exhibits greater variation in functional potential between the seep and control sites than the microbiome of the seep-sensitive *S. flabelliformis*. At the seep, the *S. flabelliformis* microbiome displays an overall loss of functional potential. In contrast, the microbiome of *C. singaporensis* shows an enhanced potential for energy-efficient carbon fixation and nitrogen metabolism, with a greater capacity to exploit excess inorganic carbon originating from the seep environment, as well as host-derived creatine. We hereby identify microbial functions potentially contributing to the success of a sponge species in naturally acidified waters.

## Results

### Microbiome overview

Shotgun metagenome sequencing revealed that both sponge species harboured microbial communities that were distinct from each other and the surrounding seawater (Supplementary Note 1 and Supplementary Fig. 1). The *C. singaporensis* microbiome was dominated by Proteobacteria, Chloroflexi and Thaumarchaeota whereas the *S. flabelliformis* microbiome was largely comprised of Proteobacteria and Thaumarchaeota. Microbial community composition varied between the control site and the adjacent CO_2_ seep site, with statistically significant differences found at multiple taxonomic levels, particularly for *C. singaporensis* (Supplementary Note 1 and Supplementary Fig. 2). The differences in microbial communities between the two species and the major shift in the composition of the *C. singaporensis* microbiome between sites confirmed previous findings^13^. However, the exclusion of Archaea from these previously published analyses precludes direct comparisons of the datasets.

**Fig. 1:**
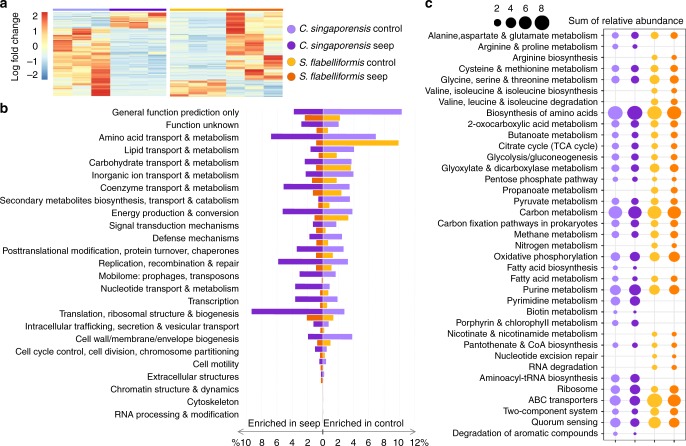
Overview of functional genes in *C. singaporensis* and *S. flabelliformis* at the CO_2_ seep and adjacent control site. **a** Heatmap with all significantly enriched genes (KOs) according to ANOVA. **b** Distribution of all genes (COGs) that were significantly enriched (ANOVA, *p* < 0.05) at the control or seep site, grouped into COG categories. Mean relative abundances (*n* = 3) were summed per COG category for each of the “enrichment groups”. **c** Relative abundance of the 30 most abundant pathways for each species (with total relative abundance >1% in control and/or seep site) based on ANOVA-derived statistically significant genes (KOs). Mean relative abundances (*n* = 3) were summed per KEGG pathway for each of the “enrichment groups”. Mean relative abundances of all genes belonging to each of the pathways were summed regardless of *p*-value

Differences between the two species were also found at the functional level (Supplementary Fig. 3, Supplementary Tables 1–2, Supplementary Note 2 and Supplementary Note 3). Functions that were more abundant at either the control or seep site, regardless of *p*-value, are hereafter termed ‘enriched’. Twenty percent of significantly enriched genes (KO annotation) in the *C. singaporensis* microbiome were more abundant at the seep, as were 80% of significantly enriched genes in the *S. flabelliformis* microbiome (Fig. 1a and Supplementary Table 1). However, genes that were enriched at the seep were generally an abundant component of the metagenome of *C. singaporensis* but a minor component of the *S. flabelliformis* metagenome. The total relative abundance of seep-enriched genes (COG annotation) was 68 and 19% for *C. singaporensis* and *S. flabelliformis*, respectively (Fig. 1b). In contrast, the total relative abundance of genes enriched at the control site was 65% and 34%, respectively. A correlation vector of environmental variables over ordination of KOs revealed that the functional profiles were primarily driven by factors associated with inorganic carbon chemistry (Supplementary Note 4, Supplementary Fig. 4 and Supplementary Tables 3–6).

### Transport of carbon and host-derived compounds

**Fig. 2:**
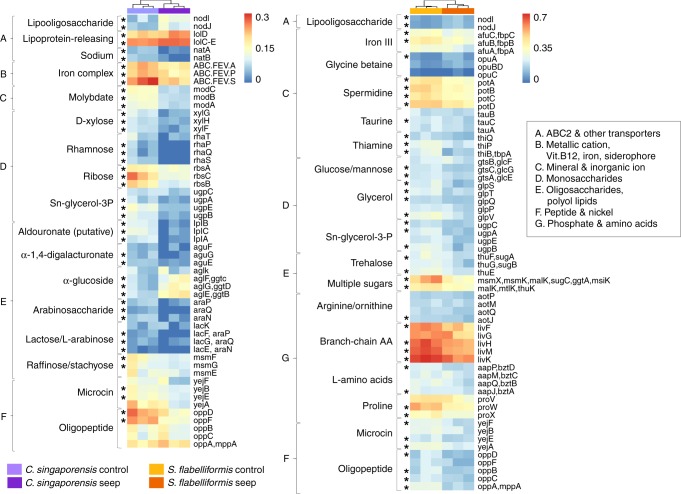
Microbial genes affecting uptake of exogenous compounds at the CO_2_ seep in *C. singaporensis* and *S. flabelliformis*. Heatmap showing genes encoding ATP-Binding Cassette (ABC) transporters significantly affected by site. Data shown as square-root transformed mean (*n* = 3) relative abundance calculated on rarefied data for each sponge species. Asterisk denotes *p* < 0.05 according to ANOVA

One of the most abundant functions at the seep in the microbiomes of both sponge species was related to ATP-Binding Cassette (ABC) transporters (Fig. 1c, Fig. 2, Supplementary Note 5, Supplementary Fig. 5 and Supplementary Fig. 6). Total organic carbon (TOC) in seawater was consistent between sites^7^ (Supplementary Table 4); however, microbiomes of both *C. singaporensis* and *S. flabelliformis* exhibited a reduced potential for uptake of carbohydrates at the seep, suggesting that the microbial community derives less benefit from external energy sources and carbon at the seep than at the control site^22^. The *C. singaporensis* microbiome exhibited a reduction in the relative abundance of genes encoding ABC importers for D-xylose (*xylFGH*, 4.9-times), ribose (*RbsABC*, 2.9-times), polysaccharides (putative transporter, *lplABC*, 15-times), alpha-1,4-digalactorunate (*aguEFG*, 6.3-times), arabinosaccharide (*araNPQ*, 5.2-times), lactose/L-arabinose (*lacEFGK*, 2-times), raffinose/stachyose (*msmEFG*, 2-times), rhamnose (*rhaPQST*, absent from the seep samples) and Sn-glycerol-3-phosphate (*ugpABCE*, 4.3-times), which provides a source of phosphate in addition to carbon^23^. In the *S. flabelliformis* microbiome, ABC transporter genes with significantly reduced relative abundance at the seep were involved in the import of glucose/mannose (*glcEFG*, 1.5-times), multiple sugars (several systems, 1.8-times), Sn-glycerol-3-phosphate (*ugpABCE*, 2.2-times), trehalose (*thuEFG*, 2-times) and the osmoprotectant glycerol (*glpPQSTV*, 2-times). The *S. flabelliformis* microbiome also exhibited reduced capacity for the uptake of several amino acids and other compounds that could serve as energy sources, with a lower relative abundance of importers for arginine/ornithine (*aotJMPQ*, 1.3-times), branched-chain amino acids (*livFGHKM*, 1.4-times), general L-amino acids (*appJMPQ*, 1.5-times), taurine (*tauABC*, 1.4-times), spermidine (*potABCD*, 1.6-times), and thiamine (*thiBPQ*, 2.3-times). Other genes related to transport and which differed between the two species are further discussed in Supplementary Note 5. The microbial communities of *C. singaporensis* and *S. flabelliformis* at the seep appear to have a restricted capacity to use exogenous organic compounds for energy production and biosynthesis, suggesting that sponge hosts, in particular *S. flabelliformis*, might retain carbohydrates and amino acids derived from filter-feeding and de novo biosynthesis, rather than providing them to the symbionts.

### Carbon fixation and carbonic anhydrase

**Fig. 3:**
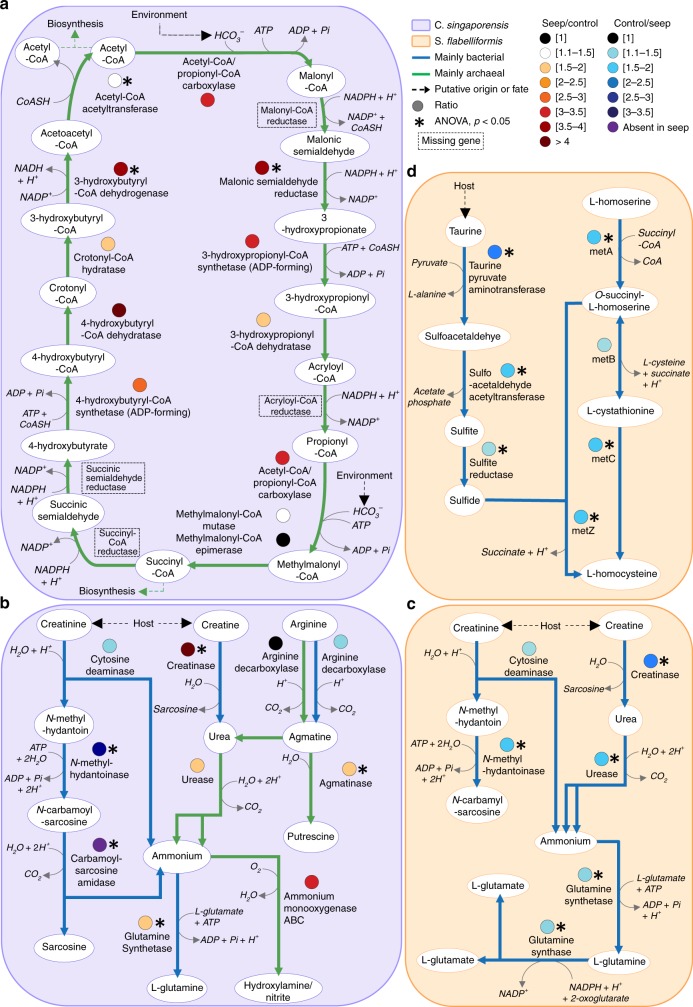
Microbial metabolic pathways related to carbon, nitrogen and sulfur metabolism. **a** Carbon fixation via HP/HB cycle (see Supplementary Methods for detail of KO assignment to functions). **b** and **c** Degradation and utilisation of nitrogen compounds.** d** Sulfide assimilation following taurine degradation. *P*-values (represented by asterisks) were derived from ANOVA. Line colours represent taxonomic origin and coloured circles represent ratios of relative abundances, all according to the colour scale described in the figure

The concentrations of bicarbonate ion (HCO_3_^−^) at Upa-Upasina were significantly higher at the seep than at the control site (1887 ± s.d 140 μmol kg^−1^ at the seep vs. 1661 ± s.d 45 μmol kg^−1^ at the control, Supplementary Table 4). An increased potential for carbon fixation via the HP/HB cycle of Thaumarchaeota was found in *C. singaporensis* at the CO_2_ seep, providing support for possible metabolic adaptation of the microbiome to the seep environment. The 3-hydroxypropionate/4-hydroxybutyrate (HP/HB) cycle typically uses bicarbonate ion to produce acetyl-coA and succinyl-coA, which can both be used for biosynthesis via further conversion into oxaloacetate, pyruvate or oxaloglutarate-CoA^24^. A two to four-times increase in the relative abundance of genes encoding key HP/HB cycle enzymes was found at the seep, including for malonic semialdehyde reductase (3.9-times, ANOVA, *p* = 0.03), 3-hydroxypropionyl-coA synthetase (3-times, ANOVA, *p* > 0.05), 3-hydroxypropionyl-coA dehydratase (1.9-times, ANOVA, *p* > 0.05), 4-hydroxybutyryl-CoA synthetase (2.8-times, ANOVA, *p* > 0.05), 4-hydroxybutyryl-CoAdehydratase (4-times, ANOVA, *p* > 0.05), 3-hydroxybutyryl-CoA dehydrogenase (3.8-times, ANOVA, *p* < 0.01) (Fig. 3a and Supplementary Fig. 8). These genes were almost all assigned to the Thaumarchaeota genus *Nitrosopumilus* (Supplementary Table 7 and Supplementary Note 13), explaining the lack of genes encoding malonyl-coA reductase, acryloyl-coA reductase, succinyl-coA reductase and succinate semialdehyde reductase, which are yet to be identified from any Thaumarchaeota^25^. *Nitrosopumilus maritimus* has been reported to use the most energy-efficient version of the HP/HB cycle described to date^26^. These findings suggest that the higher HCO_3_^−^ concentrations at the CO_2_ seep may favour energy-efficient autotrophy by Thaumarchaeota in the *C. singaporensis* microbiome.

Another potential marker for adaptation to the high bicarbonate concentrations in *C. singaporensis* at the seep was the reduced relative abundance of two genes encoding carbonic anhydrase (COG0288, 1.5-times and COG0338, 5.2-times, Supplementary Note 7 and Supplementary Fig. 9), which catalyses the interconversion between CO_2_ and HCO_3_^−^ and were primarily assigned to Cyanobacteria, Proteobacteria and Actinobacteria. Such results suggest that the increased bicarbonate concentration at the seep may reduce the selective pressure on microorganisms lacking carbonic anhydrase.

### Nitrogen metabolism

OA had a large effect on nitrogen cycling genes in the microbiomes of both species, favouring energy-efficient biochemical pathways with creatine and urease degradation in *C. singaporensis* and causing a decrease in the capacity to use key nitrogenous compounds in *S. flabelliformis* (Fig. 3b, c, Supplementary Note 8, Supplementary Fig. 10 and Supplementary Fig. 13). Sponges can produce creatine and creatinine^27^, with previous reports suggesting that these sponge-derived compounds can fuel carbon and nitrogen metabolism in symbiotic Archaea^28^. Both compounds are interconvertible via creatininase^29^, for which the encoding gene did not vary in relative abundance between sites for either species. However, genes encoding a series of enzymes involved in creatinine degradation^29^ were less abundant at the seep site for both species. In the microbiome of *C. singaporensis* these genes encoded (i) cytosine deaminase (1.4-times, ANOVA, *p* > 0.05), which hydrolyses creatinine into N-methylhydantoin with the release of a molecule of ammonia, (ii) the two subunits of N-methylhydantoinase (3.1-times and 3.4-times increase, ANOVA, both *p* < 0.01), which uses a molecule of ATP to hydrolyse N-methylhydantoin into N-carbamoylsarcosine and (iii) N-carbamoylsarcosine oxidase (absent from all seep samples), which deaminates N-carbamoylsarcosine into sarcosine and releases a second molecule of ammonia (Fig. 3b). In the *S. flabelliformis* microbiome, the genes encoding N-methylhydantoinase subunits were also significantly less abundant at the seep (1.3-time and 1.9-time, respectively, ANOVA, *p* < 0.01 for both) (Fig. 3b, Supplementary Fig. 13 and Supplementary Table 11). Simultaneously, *C. singaporensis* symbionts displayed a greater potential for creatine degradation at the seep with a 5-times increase in the relative abundance of the gene encoding creatinase (ANOVA, *p* < 0.01), which produces urea and sarcosine, and a non-significant, but 2-times increase (from 0.02% to 0.04%) in genes encoding all urease subunits and accessory proteins, which release two molecules of ammonia and one molecule of CO_2_, without using ATP. *C. singaporensis* thus might preferentially transfer creatine to its symbionts over creatinine at the CO_2_ seep, supporting our findings that energy conservation is an important feature of successful sponge-microbes symbiosis in an acidified ocean.

In contrast, the *S. flabelliformis* microbiome exhibited a decreased potential for creatine and urea degradation, as well as glutamine and glutamate biosynthesis at the CO_2_ seep, a decrease in genes encoding creatinase (2.4-times, EC 3.5.3.3, ANOVA, *p* < 0.01), all urease subunits and accessory proteins (1.6-times on average, ANOVA, *p* < 0.05 except for *ureE* and *ureJ*), glutamine synthetase, which assimilates one molecule each of ammonia, glutamate and ATP into glutamine (1.3-times, ANOVA, *p* < 0.01), and glutamate synthase, which leads to glutamate (1.4-times, ANOVA, *p* < 0.01) (Fig. 3c). It therefore appears that at the CO_2_ seep, *S. flabelliformis* symbionts have reduced capacity to benefit from host-derived creatine and creatinine fuelling nitrogen metabolism. While experimental work is still required to confirm nutrient fluxes, this diminished capacity for nitrogen metabolism likely contributes to the apparent sensitivity of *S. flabelliformis* to acidified waters.

The central role of Archaea in the microbiome of *C. singaporensis* under OA was further confirmed by interrogating the taxonomic origin of the genes involved in nitrogen cycling. Creatine degradation genes were exclusively assigned to bacterial scaffolds in both species (Supplementary Table 8), except for the urease subunits in *C. singaporensis*, which were primarily archaeal, and the ratio of archaeal to bacterial scaffolds for these genes increased at the CO_2_ seep. Accordingly, a greater potential for urea production by the archaeal community was identified in the *C. singaporensis* microbiome at the seep via agmatine degradation (Fig. 3b). Specifically, a significantly higher relative abundance of the agmatinase-encoding gene (1.6-times, EC 3.5.3.11, ANOVA, *p* = 0.01), responsible for converting agmatine into urea and putrescine, was found. Whilst this gene was detected on both bacterial and archaeal scaffolds in *C. singaporensis* at the control site, it was more prevalent on archaeal scaffolds at the seep site (Supplementary Table 8). Agmatine is produced via arginine decarboxylase, for which we found two encoding genes: *speA* (K01585), which was primarily carried by bacterial scaffolds and was consistent across sites (ANOVA, *p* > 0.05), and *pdaD* (K02626), which was primarily found on archaeal scaffolds and doubled in relative abundance at the seep (0.011% to 0.024%, ANOVA, *p* > 0.05). Archaeal genes for glutamate-supported arginine biosynthesis were also detected in *C. singaporensis* at the seep (Supplementary Fig. 11, Supplementary Fig. 12, Supplementary Table 9 and Supplementary Table 10). The ammonia produced as a central component of the cascade could be used by Archaea for ammonia oxidation^30^, as we found at the seep a 3-times increase in the relative abundance of genes encoding ammonia monooxygenase (*amoABC* cluster, ANOVA, *p* > 0.05), and scaffolds carrying this gene cluster were primarily assigned to Thaumarchaeota (100% at the seep and 74% at the control) (Supplementary Table 8). It could also serve the microbial community of *C. singaporensis* for assimilation, which is consistent with the significant increase in the gene encoding glutamine synthetase at the seep (1.3-times, ANOVA, *p* < 0.01).

Together these results indicate that at the control site ammonia production via urea is predominantly supported by bacterial-driven creatinine metabolism, whereas archaeal-driven arginine metabolism and energy-efficient bacterial-driven creatine catabolism predominates in the *C. singaporensis* microbiome at the seep site.

### Sulfur metabolism

OA negatively impacted the metabolic potential for taurine dissimilation and sulfur assimilation in *S. flabelliformis*, which may reflect reduced host production of taurine in acidified seawater. Indeed, taurine can represent a considerable proportion of total free amino acids in sponges^31^ and the microbiome of the seep-sensitive species presented a significant decrease in the relative abundance of genes encoding three enzymes in the taurine degradation pathway:^32^ taurine-pyruvate aminotransferase (*tpa*, 2.3-times, ANOVA, *p* < 0.01), which degrades taurine into sulfoacetaldehyde and L-alanine, sulfoacetaldehyde acetyltransferase (*xsc*, 1.6-times, ANOVA, *p* < 0.01), which produces sulfite and acetate phosphate, and *cysI* (1.4-times, ANOVA, *p* = 0.02), which converts sulfite into sulfide (Fig. 3d and Supplementary Fig. 14). Most of the scaffolds carrying *tpa* had sequence similarity to Thiotrichales and unclassified Gammaproteobaceria, corroborating the taxonomic results (Supplementary Fig. 2, Supplementary Table 12 and Supplementary Note 9). Scaffolds carrying *xsc* were assigned to diverse taxa, as sulfoacetaldehyde can also result from the decarboxylation of sulfopyruvate in the sulfolactate degradation pathway. The bacterial orders Burkholderiales (Betaproteobacteria) and Oceanospirillales (Gammaproteobacteria) represented a lower proportion of the taxa carrying the *xsc* gene at the seep and therefore appear to be primarily responsible for the decline in relative gene abundance of *xsc* (Supplementary Table 12).

*S. flabelliformis* microbiomes also exhibited a lower potential for sulfur assimilation at the seep, as genes composing two pathways for the conversion of L-homoserine into L-homocysteine were significantly less abundant at the CO_2_ seep (Fig. 3c). Three of the four genes encoding these pathways’ enzymes, namely homoserine O-succinyl-transferase (*metA*), cystathionine-β-lyase (*metC*) and O-succinyl-homoserine sulfydrylase (*metZ*), were significantly less abundant at the seep, with 1.9-times, 1.6-times and 1.5-times changes, respectively (ANOVA, *p* < 0.01, *p* = 0.01 and *p* < 0.01 respectively) (Fig. 3c). Taxa potentially contributing to these pathways include the orders Burkholderiales (Betaproteobacteria), Methylococcales (methanotrophic Gammaproteobacteria), Oceanospirillales (heterotrophic Gammaproteobacteria) and Chromatiales (anoxygenic photosynthetic Gammaproteobacteria) (Supplementary Table 12). Ocean acidification thus reduces the potential for the *S. flabelliformis* microbiome to use taurine to produce sulfide and subsequently assimilate it into biomass. Whether driven by pH-induced reductions in host taurine production or through a reduced potential for microbial dissimilation, this reduced capacity for sulfur metabolism could contribute to the poor performance of *S. flabelliformis* in acidified waters.

## Discussion

Our study characterises the potential effects of OA on marine microbial symbioses. However, metagenomic sequencing has several limitations that need to be acknowledged. Whilst differences in the relative abundance of specific pathways indicate a shift in functional potential within the microbiome, these pathways need to be expressed to effectively alter metabolism. For example, despite considerable evidence that Thaumarchaeota oxidise ammonia and emerging research confirming they also metabolise urea^33^, it is possible that increased relative abundances of these pathways within the *C. singaporensis* microbiome do not translate into changes in metabolism. Future studies should therefore utilise complimentary transcriptomic, proteomic, metabolomic approaches or stable isotope probing to unequivocally validate altered symbiont metabolism under OA. Similarly, we acknowledge limitations in using results derived from CO_2_ seeps for inferring impacts under future acidified oceans. Firstly, changes in oceanic carbonate chemistry will occur concomitantly with other stressors including ocean warming, pollution, overfishing and habitat destruction, with the potential for additive, mitigating or synergistic effects^34^. However, the Upa-Upasina location is not exposed to anthropogenic pressures such as pollution or eutrophication and occasional mixing of the water column during high-wind seasons means that organisms may experience periods of respite from low pH conditions at the seep^5^. Secondly, in addition to changes in functional potential of the microbiome, other factors such as habitat structure, competition for space, predation, host metabolism, reproductive success or larval settlement rates could contribute to the differential abundance of the two sponge species across sites, particularly the striking 40-times increase in *C. singaporensis* at the seep. Additional information on ecological and physiological characteristics of *C. singaporensis* is now needed to tease apart the relative contributions of each of these factors in the success of this species at the Upa-Upasina seep.

**Fig. 4:**
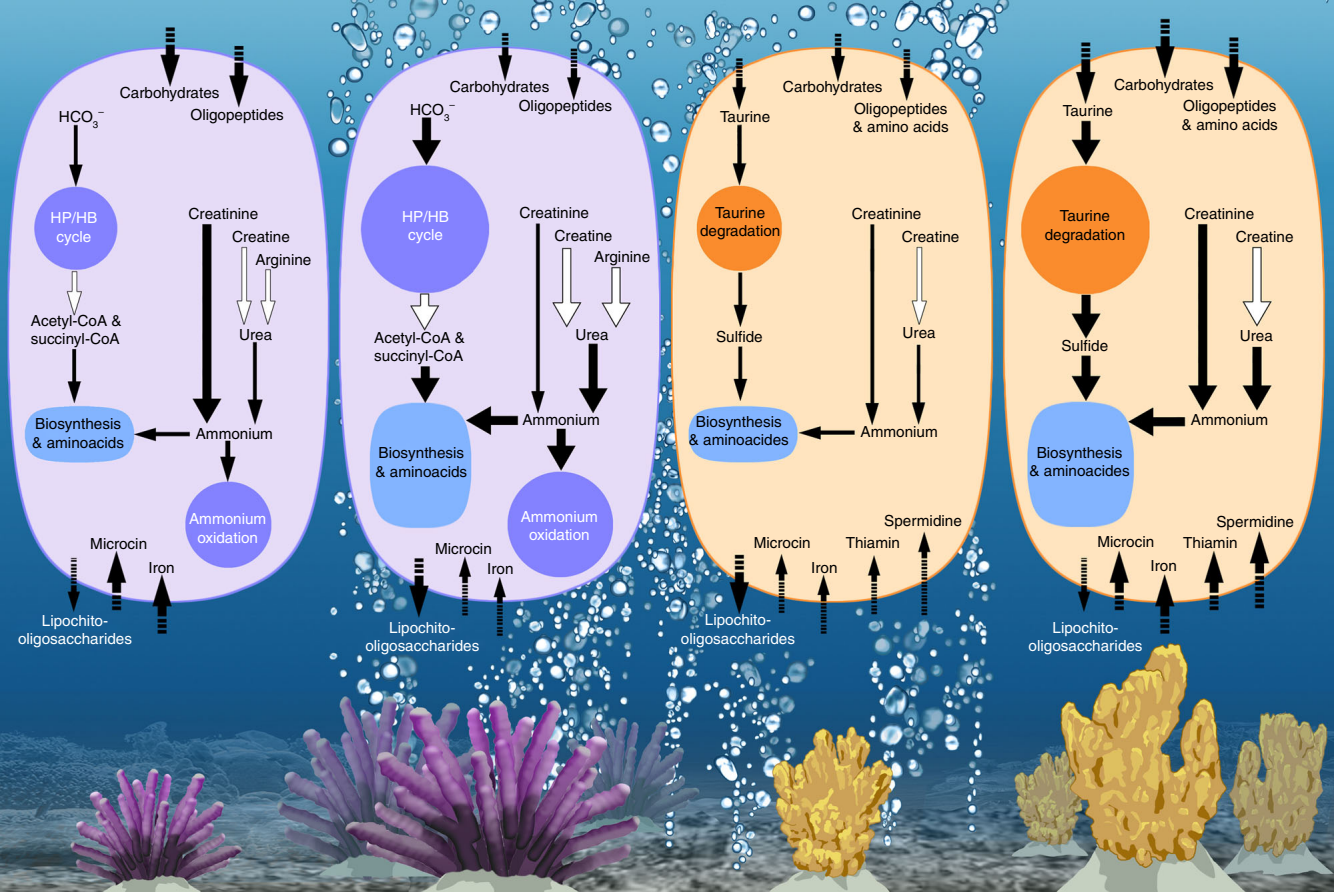
Schematic summary of the functions affected by the CO_2_ seep in microbiomes of *C. singaporensis* (represented in purple) and *S. flabelliformis* (represented in orange)

Mindful of these considerations, we nevertheless uncover an enhanced functional potential for energy-efficient carbon and nitrogen metabolism in a sponge species that is tolerant of OA conditions projected for 2100 and show a general loss of functional potential in an OA-sensitive species (Fig. 4). Host metabolic adaptation has been detected as a stress response of sponges under OA^35^ and reported as a survival mechanism for invertebrate species at CO_2_ seeps^36^. However, rapid rates of microbial evolution, symbiont switching/shuffling (i.e., community reorganisation) and vertical transmission of symbionts across generations can also contribute to microbiome-mediated adaptation^37^. Multicellular organisms can maintain homoeostasis under OA by altering energy allocation for different biological processes^38–40^. Remarkably, a similar compensatory mechanism might also be occurring at the microbial scale, with the microbiome of OA tolerant species enriched in energy-efficient pathways that could enable excess energy to be utilised elsewhere as needed. Given the key roles that microbes play in host health^18^, metabolic adaptation of the microbiome could also confer tolerance to future acidified oceans.

## Methods

### Sample collection

Samples were collected in the Milne Bay province of Papua New Guinea at Upa-Upasina in March 2014 following published methods^13^ and as part of Expedition 3^7^. In detail, three biological replicates of *C. singaporensis* and *S. flabelliformis* were collected on SCUBA at the seep and control sites (500 m apart) and brought back to the surface in individual plastic bags filled with seawater from the collection point. Small tissue samples from each specimen were preserved in 70% ethanol for species identification and the remaining tissue was snap-frozen for microbial enrichment and metagenomic analysis. Twelve litre seawater samples were also collected in triplicate from the control and seep sites and successively filtered through 5 μm and 0.22 μm Sterivex filters, which were subsequently stored at −20 °C until processing. All samples were transported to the Australian Institute of Marine Science according to AQIS regulations (permit number 14002493). Sponge species were identified on the basis of morphological characters^41^ by taxonomic specialists at Western Australian Museum and conformed to original descriptions for *C. singaporensis*^42^ and *S. flabelliformis*^43^.

### Sample processing and sequencing

Microbial cells were enriched following established procedures^44^ with minor modifications. In detail, sponge tissue samples transferred to sterile Falcon tubes (Corning, New York, USA) were rinsed twice in a shaker set to 200 rotations per minute (rpm) for 5 min in ~10 mL of sterile Calcium/Magnesium-Free Sea Water (CMFSW) containing 0.4 M NaCl, 10 mM KCl, 7 mM Na_2_SO_4_ and 0.05 mM NaHCO_3_ in milliQ (mQ) water. The rinsed tissue was cut into small (~ 1 cm^3^) pieces using a 70% ethanol-rinsed and CMFSW-rinsed Tupperware TurboChef (Orlando, Florida, USA) and homogenised in ~10–12 mL of CMFSW for a maximum of 10 min with a SilentCrusher M homogeniser (Heidolph, Schwabach, Germany). All instruments and equipment used were thoroughly washed with 70% ethanol and CMFSW between samples. Sterile collagenase (Sigma-Aldrich, St Louis, Missouri, USA) diluted into sterile CMFSW was added to each sample at a concentration of 0.5 g L^−1^ before incubation at 150 rpm for 30 min. Each sample was filtered through a 100 μm Nylon sterile cell strainer (Corning, New York, USA) and centrifuged for 15 min at 100 × *g* at 4 °C. The supernatant collected into sterile tubes was centrifuged for 15 min at 300 rpm at 4 °C. The supernatant was again centrifuged for 15 min at 300 rpm at 4 °C. The newly formed supernatant was filtered twice through sterile 8 μm TETP02500 Isopore membrane filters (Merck, Darmstadt, Germany) and twice through sterile 5 μm TMTP02500 Isopore membrane filters (Merck, Darmstadt, Germany). The microbial fraction of the samples was pelleted with a centrifugation at 8000* × g* for 20 min at 4 °C. The pellet was rinsed in ~5–10 mL of a sterile buffer containing 10 mM Tris HCl at pH 8 and 0.5 M NaCl, and centrifuged again at 5000* × g* for 20 min at 4 °C. This cell wash was repeated twice. A final centrifugation was conducted at 5000 × *g* for 20 min at 4 °C. The resulting concentrated microbial pellet was recovered in 1 mL of sterile Tris-HCl-NaCl at pH 8 and aliquoted into 200 μL sterile tubes stored at −20 °C. To minimise carry-over of potential eukaryotic DNA resulting from possible lysis of eukaryotic cells during the microbial enrichment process, a DNase treatment was conducted immediately prior to DNA extraction using DNase I from Epicentre (Illumina, Madison, Wisconsin, USA), according to the manufacturer’s instructions and followed by DNase I inactivation via incubation at 65 °C for 10 min. DNA was extracted using the UltraClean kit (MO BIO, San Diego, USA) according to the Manufacturer’s instructions. Extracted DNA was further purified using the ZymoResearch (Irvine, California, USA) purification kit (Genome DNA clean and concentrator). DNA from seawater was extracted directly from Sterivex filters^45^ as follows: 2 mL capacity Sterivex filter bells (Merck, Darmstadt, Germany) were filled with 1.8 mL of a lysis buffer containing 40 mM EDTA, 50 mM Tris-HCl at pH 8 and 0.75 M sucrose and sealed, then incubated with gentle rotation at 37 °C for 45 min. Two-hundred microliters of 2 mg mL^−1^ proteinase K in 10% SDS were added to each filter bell which was then incubated with gentle rotation at 55 °C for 1 h. The lysate was recovered in Eppendorf tubes and received equal volumes of phenol:chloroform:IAA (25:24:1, pH 8.0). The mixture was centrifuged at 16,000 × *g* for 5 min. The top layer was transferred to a fresh tube and received equal volumes of chloroform:IAA (24:1), before centrifugation at 16,000 × *g* for 5 min. The DNA contained in the supernatant was precipitated in a fresh tube by adding 0.1 volume sodium acetate 3 M and 0.6 volume 100% isopropanol and incubating for 10 min at room temperature. The DNA was pelleted by centrifugation at 16,000 × *g* for 30 min. After removing the supernatant, 1 mL of 70% ethanol was added. A final centrifugation at 16,000 × *g* for 15 min was conducted. Ethanol was removed and left to completely evaporate. The clean DNA was resuspended in 20 μL of mQ water and left at 4 °C overnight, then stored at −20 °C. DNA quality and concentrations were checked using Nanodrop and Qubit. Nextera XT library preparation was performed on all samples at the Ramaciotti Centre for Genomics (University of New South Wales, Australia) before multiplexing and sequencing across four lanes of Illumina MiSeq (2 × 250 bp).

### Metagenomes assembly

Raw reads were quality filtered with Prinseq^46^ to discard sequences with ambiguous bases or those that were shorter than 30 bp. Reads were assembled using IDBA-UD with default parameters and the pre-correction option^47^. Reads were mapped back to the assembly with Bowtie2^48^ to assess assembly quality and obtain coverage information. Statistics collected during the assembly process are provided in Supplementary Table 18. Raw reads were submitted to the Sequence Read Archive [https://www.ncbi.nlm.nih.gov/sra] and can be found under SRA accession number SRP159543 (Supplementary Table 19). Metagenome-assembled genomes (MAGs) were binned according to Supplementary Methods and used to confirm specific results (Supplementary Note 13 and Supplementary Table 20).

### Taxonomic assignment

Taxonomy was assigned to 16S rRNA subunit genes extracted from the metagenomic assemblies using GraftM^49^. Taxonomic resolution at the species level was not always possible, resulting in some scaffolds only being assigned to higher ranks. GraftM packages can be found at [https://data.ace.uq.edu.au/public/graftm/7/].

### Functional annotation

Scaffolds were submitted to IMG/MER^50^ for annotation along with coverage information to enable the estimation of gene copy numbers. Scaffolds identified by IMG to be of Eukaryotic origin were removed. Estimated copy numbers of Clusters of Orthologous Genes (COGs) and KEGG Orthology (KO) terms were retrieved from the KEGG database^51^ after annotation through the IMG pipeline using the “standard gene calling methods” protocol from IMG^52^. Counts of individual COGs and KEGGs were used for statistical analyses. Analysis of the COGs and KOs was performed over three stages: (i) for each species, KOs which were significantly different between sites (identified as described under “statistical analysis”) were clustered in a heatmap (Fig. 1a). The same method was applied to COGs, which were subsequently grouped into COG categories (Fig. 1b). COGs which were assigned to several categories were not “dereplicated” but left in all categories assigned; (ii) significantly enriched KOs were grouped into KEGG pathways to identify the thirty most abundant pathways for each species (Fig. 1c). As non-significant differences could be biologically meaningful, the average relative abundance of all KOs belonging to a pathway were summed for each site to assess the portion of the metagenome represented by these thirty pathways. Pathways with a total relative abundance over 1% were retained. Detailed analyses presented in Fig. 2 and Fig. 3 were performed on functions previously reported as important for sponge symbiosis^18,20,53–55^, OA and pH-related functions^9,56,57^ and functions identified as being abundant or greatly affected by site. KEGG orthology lists were compared to the IMG-derived KO list for each species. Pathways of interest were manually checked using the MetaCyc database^58^ and relevant literature (See Supplementary Methods). Taxonomic assignment of scaffolds utilised the IMG data. Additional results not discussed in the main paper, were obtained following the same procedure, including results related to eukaryotic-like proteins, (Supplementary Fig. 7 and Supplementary Note 6), sulfur metabolism (Supplementary Figs. 15–16, Supplementary Tables 13–14, and Supplementary Note 10) and vitamin production (Supplementary Figs. 17–18, Supplementary Tables 15–17 and Supplementary Notes 11–12).

### Statistical analysis of biological data

Rarefaction curves were generated for taxon and gene datasets to assess differences in coverage between samples and for quality control. Bray-Curtis similarity matrices were generated for genes (KOs) using the R package Vegan^59^. Variation amongst samples was visualised using hierarchical clustering, as well as Principal Coordinate Analysis using the “PERMANOVA+ for Primer” software package^60^ (Supplementary Fig. 3). Cluster analysis confirmed clear separation between seawater and sponge samples and greater partitioning between control and seep samples for sponges compared to seawater. A Permutational Multivariate Analysis of Variance (PERMANOVA) was performed on the Bray-Curtis matrix of KOs and showed an interaction between site and species in the partitioning of the samples (*p* = 0.0237, Supplementary Table 2), which enabled us to perform a more constrained analysis of microbial functions in relation to environmental variables (see section hereafter). Given the focus of this manuscript, no further analyses were performed on the seawater samples. Gene copy numbers were rarefied to the lowest number across samples for each species to account for differences in sequencing depth. Mean and standard deviations were calculated on percentage relative abundance of rarefied data per species. Counts of taxa and genes were modelled using negative binomial generalised linear models with site (control vs. seep) as a fixed explanatory covariate and the total gene copy number as an offset term. Any taxon/gene present in less than two samples was discarded prior to creating the models. ANOVA was used to test for significance of condition, with p-values adjusted for multiple testing using the false discovery rate. Adjusted p-values < 0.05 were used to select taxa and genes that differed in abundance between control and seep environments. Model estimates were generated using an offset = 100, which provided data on a scale representing relative abundances i.e., estimated mean count per 100 counts. These procedures were conducted using the R programme^61^ [www.R-project.org/] with R studio^62^ [https://www.rstudio.com/] and packages Stats^63^ and CAR^64^. Data handling and graphics were produced using the following R packages: tidyr, dplyr, tibble and ggplot2^65^ [http://tidyr.tidyverse.org], as well as matrixStats^66^ [https://cran.rstudio.com/web/packages/matrixStats/index.html] and RColorBrewer^67^ [https://cran.r-project.org/web.packages/RColorBrewer/index.html].

### Statistical analysis of environmental data

The control and seep sites at the Upa-Upasina location exhibit near-identical temperatures (annual averages of 28.69 °C ± 1.37 and 28.82 °C ± 1.38, respectively) and salinities (annual averages of 35.01 ppt ± 0.63 and 34.77 ppt ± 0.54, respectively). Metrics for the complete suite of environmental variables and water chemistry recorded from the Upa-Upasina site are shown in Supplementary Tables 3–5, along with results from individual *t*-tests performed on each of these variables to determine any significant difference between sites.

### Microbial functions in relation to environmental variables

To determine the role of environmental variables in driving the partitioning of samples between sites, the mean values derived from twenty-one environmental parameters were assessed against the mean Bray-Curtis matrix of KOs using a canonical analysis of principle coordinates (CAP). Mean values of KOs, within species and sites, were used for the analysis to align with environmental data derived at the site level. The CAP function in PRIMER v7^60^ performed distance-based canonical correlation of the KOs to the environmental data, thereby constraining the ordination according to variables most significantly driving differences between samples (Supplementary Note 4, Supplementary Fig. 4 and Supplementary Table 6).

### Reporting summary

Further information on research design is available in the Nature Research Reporting Summary linked to this article.

## Supplementary information

Supplementary Information

Reporting Summary

Source Data

## Data Availability

Raw reads and MAGs are available from the NCBI SRA under accession number SRP159543. Assemblies (see Supplementary Table 18 for assemblies ID) are available from IMG/MER [https://img.jgi.doe.gov/cgi-bin/m/main.cgi]. The source data underlying all figures and Supplementary Figures, except Supplementary Fig. 11 are provided as a Source Data file.
