# Supplementary material for: Changes in the metabolic potential of the sponge microbiome under ocean acidification
Source: Nat Commun. 2019 Sep 12;10:4134. doi: 10.1038/s41467-019-12156-y (PMC6742649; doi:10.1038/s41467-019-12156-y)
Supplement: Supplementary file 1 — Supplementary Information [file 41467_2019_12156_MOESM1_ESM.pdf]

Supplementary Information

Changes in the metabolic potential of the sponge microbiome under ocean acidification

Botté et al.

Supplementary Figures

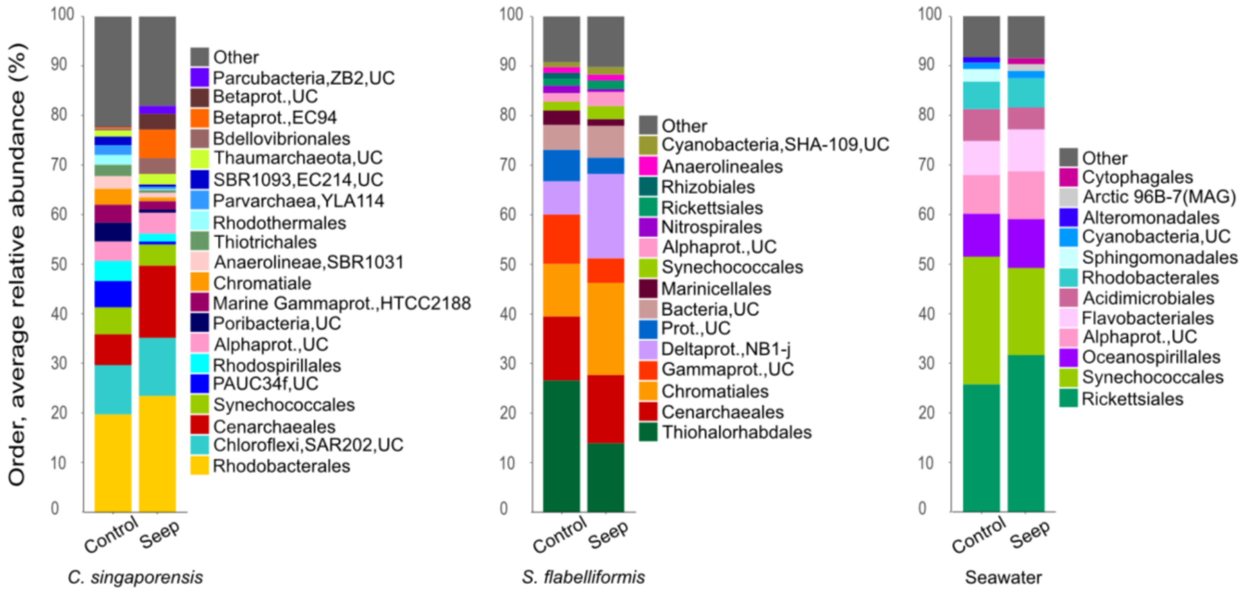

Supplementary Figure 1. Taxonomic composition of the sponge microbiome, summarised at the taxonomic rank of Order. Classification derived from GraftM analysis. Only taxa with relative abundances >1% in either control or seep are shown. UC: Unclassified; prot.: proteobateria; Gemmat.: Gemmatimonadetes, MAG: Marine Group A.

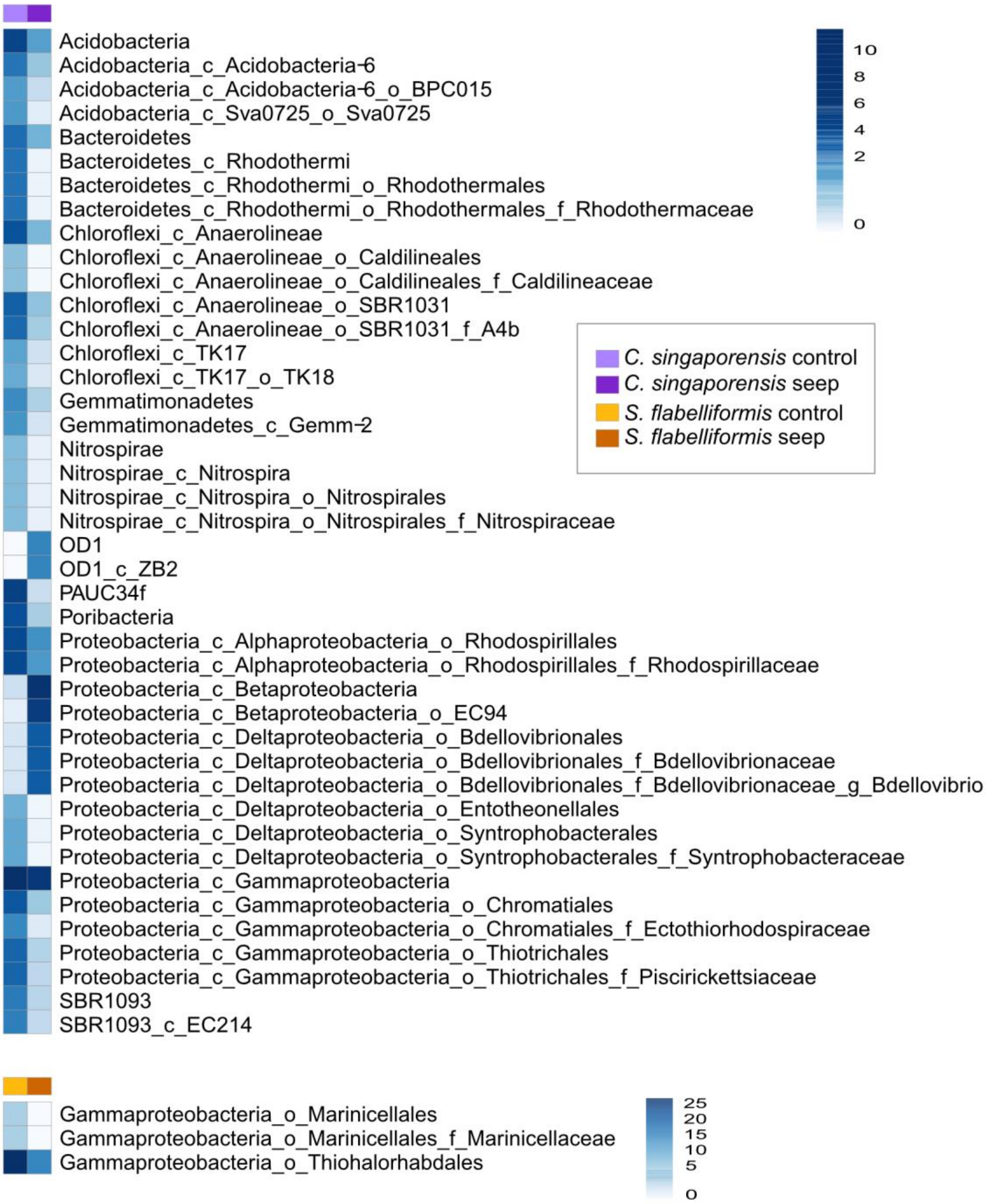

Supplementary Figure 2. Taxa with significantly different abundances between sites. Heatmaps show relative abundance. Classification derived from GraftM. Only taxa with an abundance > 1% are shown. Statistical significance was derived from ANOVA. Source data are provided as a Source Data file.

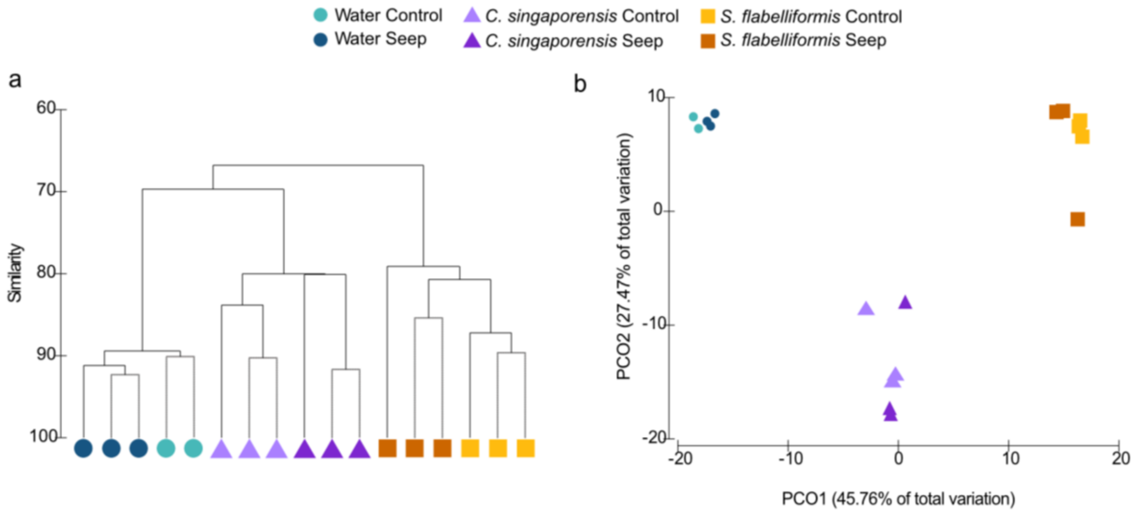

Supplementary Figure 3. Variation between replicates and sample types based on functional genes (KO) visualised as hierarchical clustering **(a)** and Principal Component Analysis **(b)** based on Bray-Curtis resemblance matrix with rarefied relative abundance as raw data.

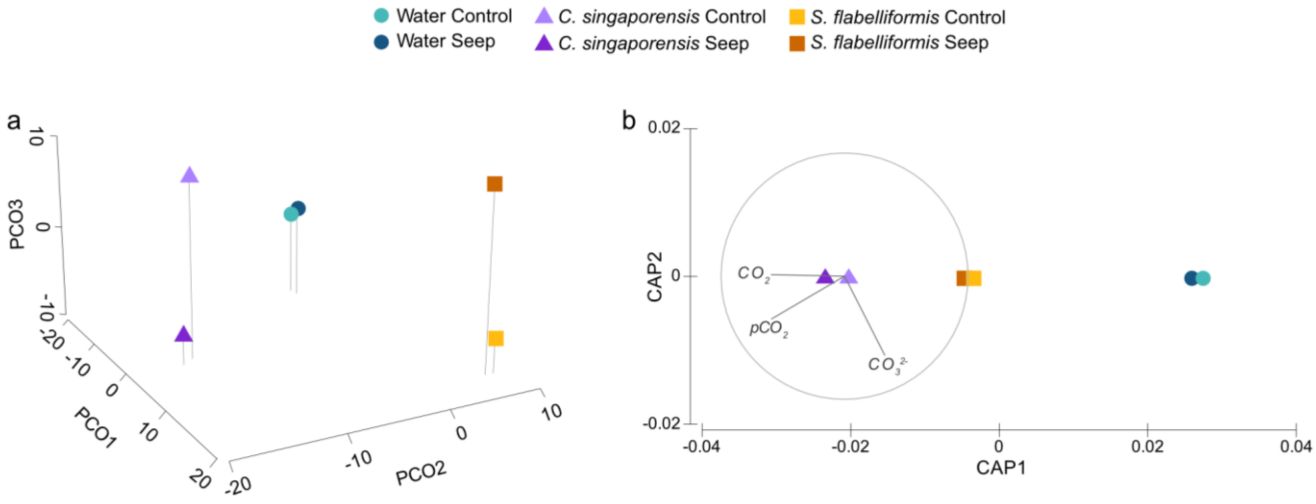

Supplementary Figure 4. Partitioning of samples based on Bray-Curtis resemblance matrix of the mean of rarefied relative abundance of functional genes (KO) per species, visualised as **(a) Principal Component Ordination** with 3 axes and **(b) Canonical Analysis of Principal Coordinates** showing environmental variables with R > 0.5 as vectors.

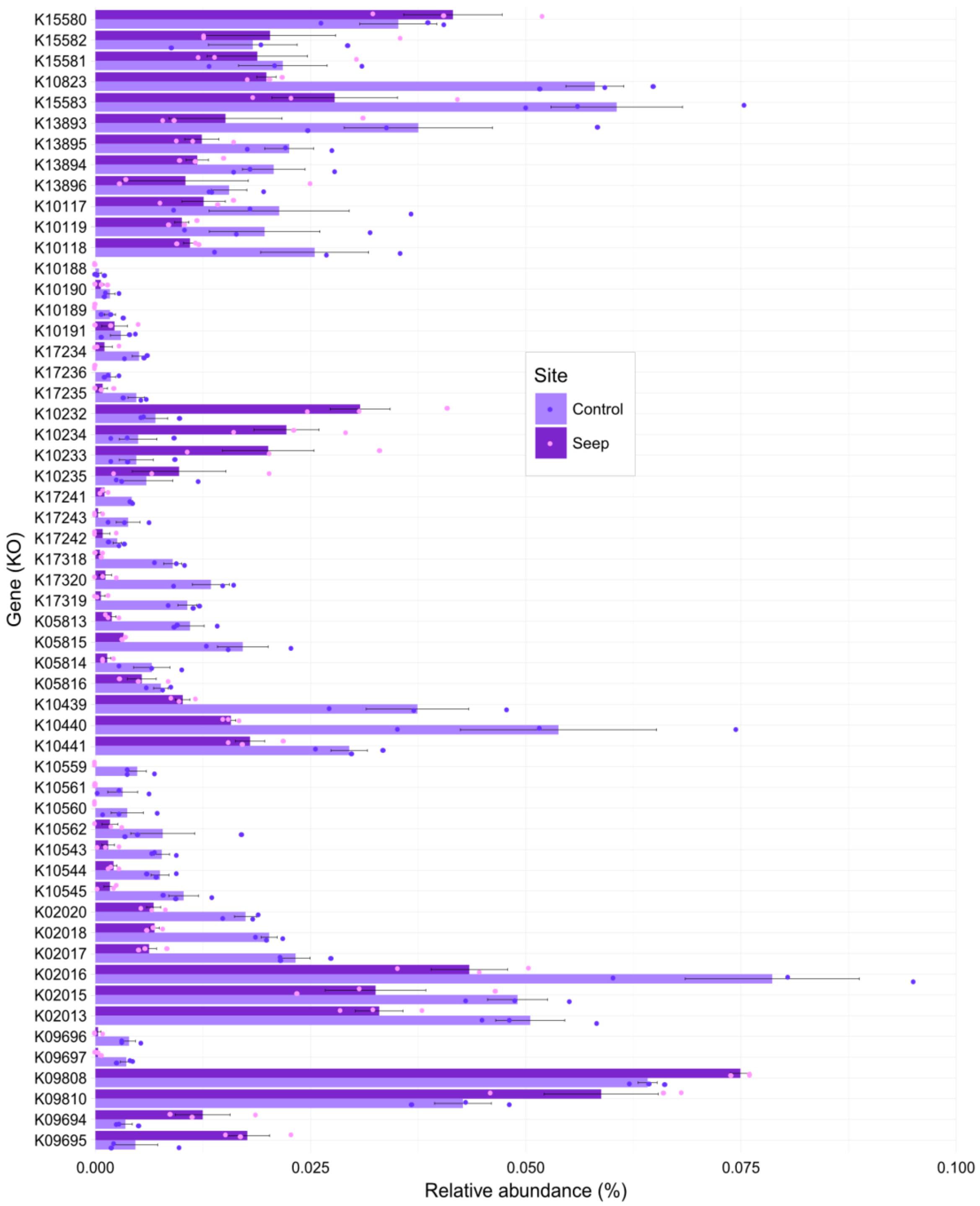

Supplementary Figure 5. Functions involved in import and/or export of molecules and compounds in *C. singaporensis*. Mean (n = 3 ± s.e.m) relative abundance calculated on rarefied counts and expressed as a percentage. Source data are provided as a Source Data file.

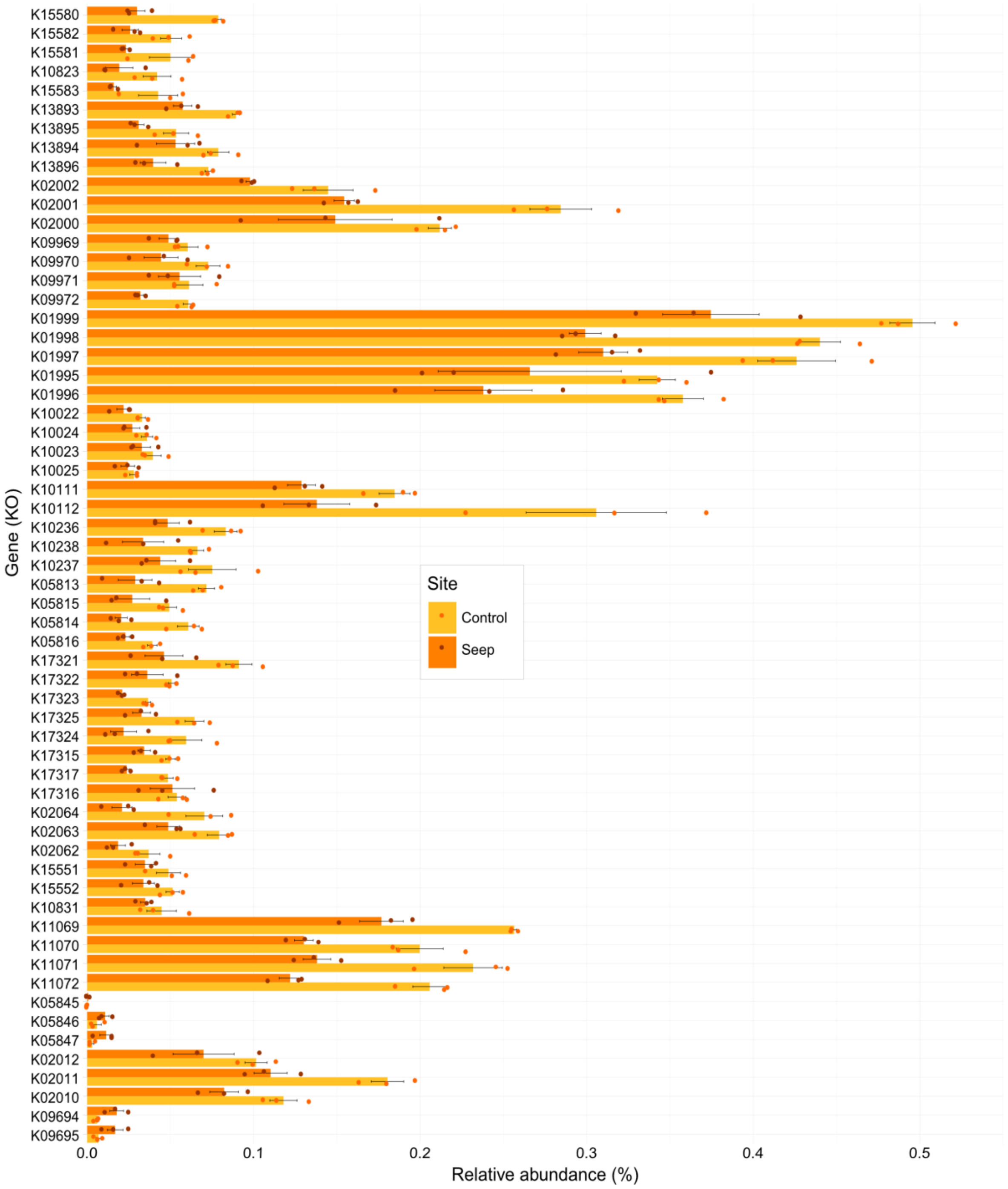

Supplementary Figure 6. Functions involved in import and/or export of molecules and compounds in *S. flabelliformis*. Mean (n = 3 ± s.e.m) relative abundance calculated on rarefied counts and expressed as a percentage. Source data are provided as a Source Data file.

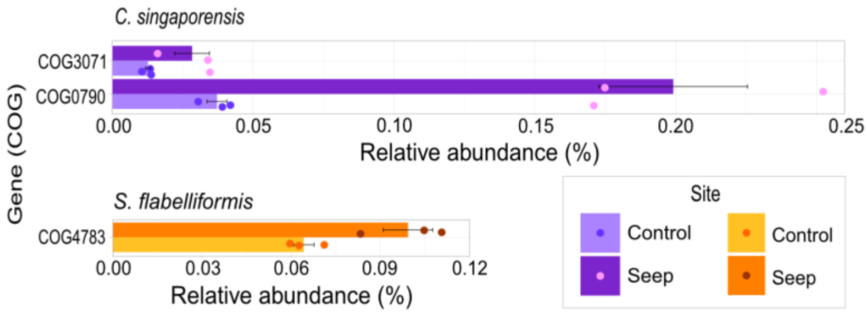

Supplementary Figure 7. Functions encoding Eukaryotic-Like Proteins in *C. singaporensis* and *S. flabelliformis* metagenomes. Mean (n = 3 ± s.e.m) relative abundance calculated on rarefied counts and expressed as a percentage. Source data are provided as a Source Data file.

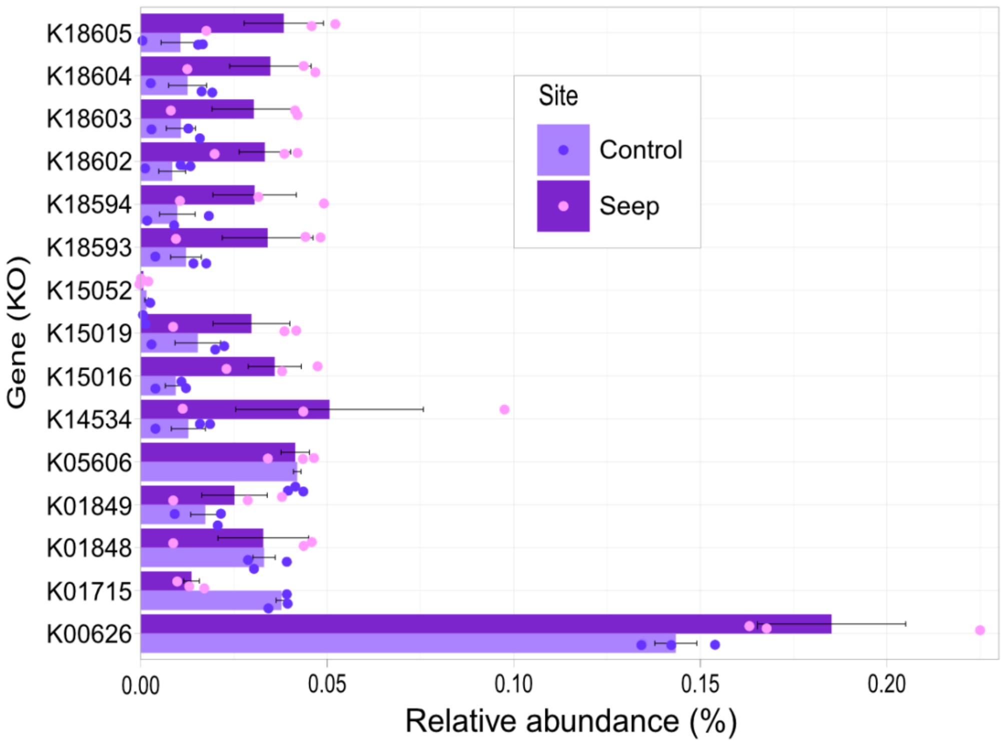

Supplementary Figure 8. Functions (KOs) involved in the HP/HB cycle in *C. singaporensis* metagenomes. Mean (n = 3 ± s.e.m) relative abundance calculated on rarefied counts and expressed as a percentage. Source data are provided as a Source Data file.

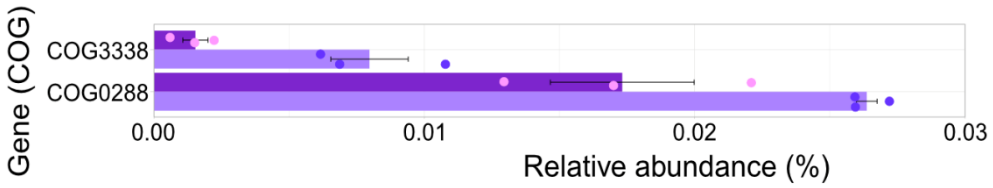

Supplementary Figure 9. Functions (COGs) encoding carbonic enhydrase in *C. singaporensis* metagenomes. Mean (n = 3 ± s.e.m) relative abundance calculated on rarefied counts and expressed as a percentage. Source data are provided as a Source Data file.

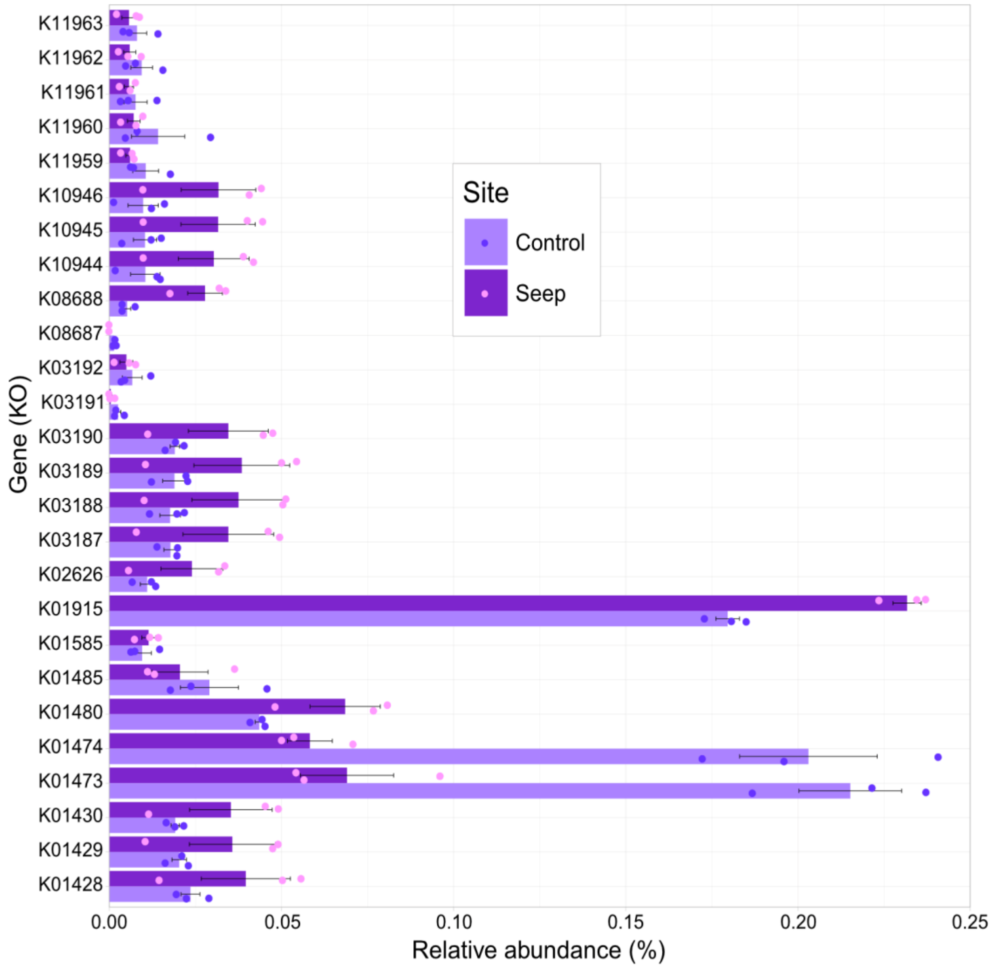

Supplementary Figure 10. Functions involved in creatine and creatinine degradation, arginine-driven urea production and urea degradation in *C. singaporensis*. Mean (n = 3 ± s.e.m) relative abundance calculated on rarefied counts and expressed as percentage. Source data are provided as a Source Data file.

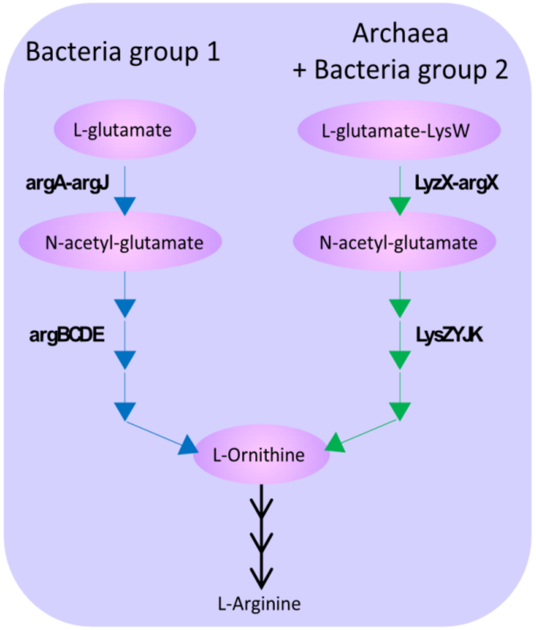

Supplementary Figure 11. Schematic of simplified arginine biosynthesis in Bacteria and Archaea.

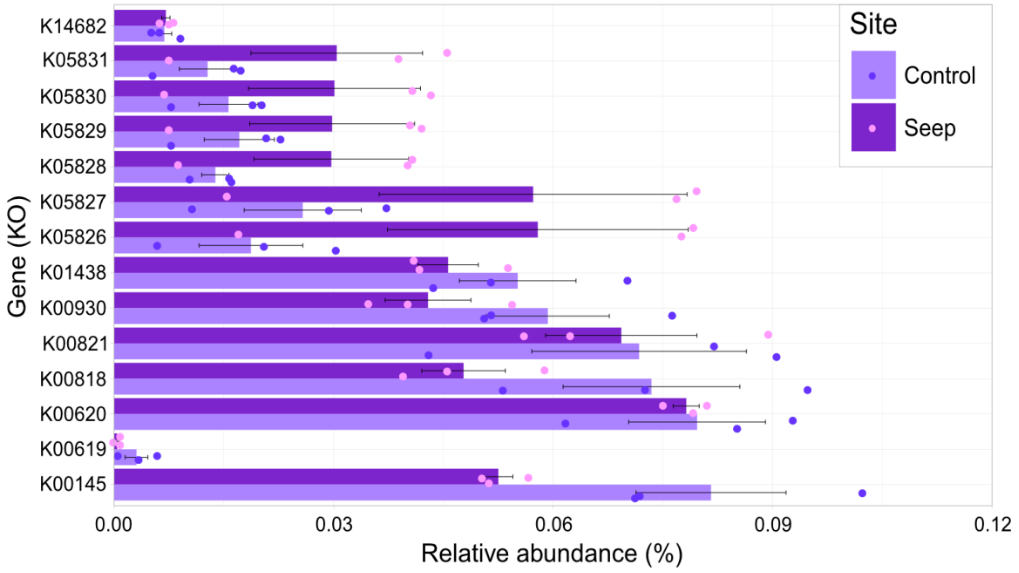

Supplementary Figure 12. Functions involved in arginine biosynthesis in *C. singaporensis*. Mean (n = 3 ± s.e.m) relative abundance calculated on rarefied counts and expressed as percentage. Source data are provided as a Source Data file.

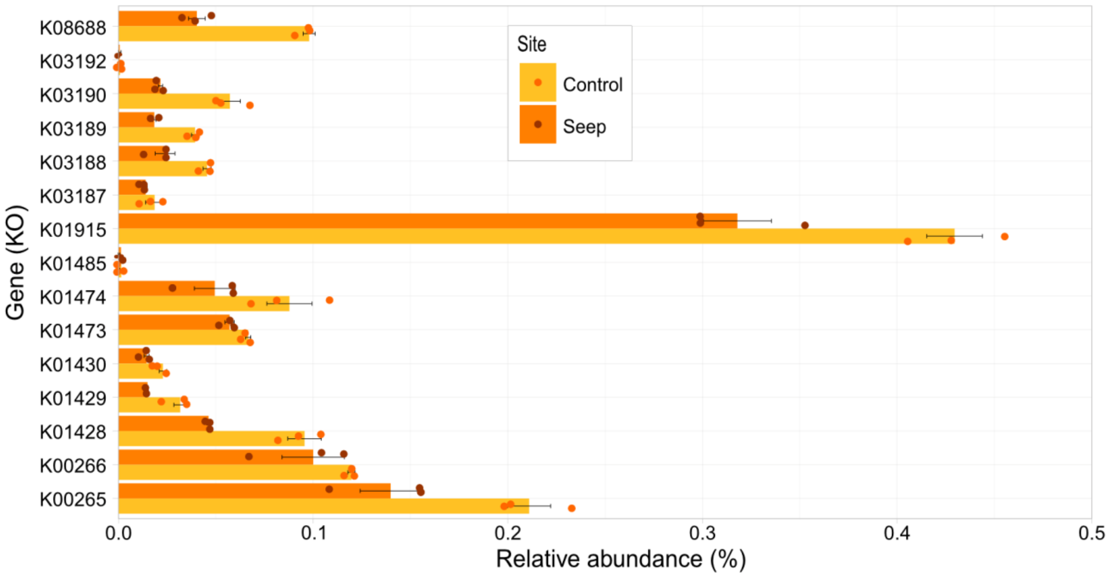

Supplementary Figure 13. Functions involved in creatine and creatinine degradation, urea production and urea degradation in *S. flabelliformis*. Mean (n = 3 ± s.e.m) relative abundance calculated on rarefied counts and expressed as a percentage. Source data are provided as a Source Data file.

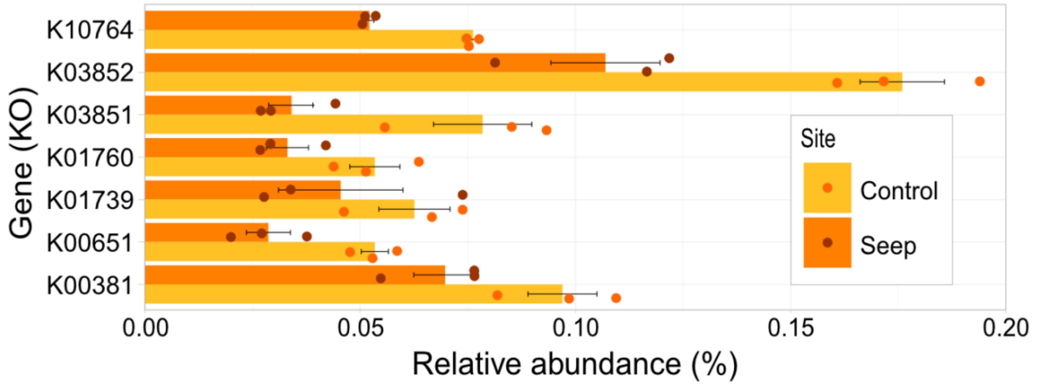

Supplementary Figure 14. Functions involved in taurine degradation and sulfide assimilation in *S. flabelliformis*. Mean (n = 3 ± s.e.m) relative abundance calculated on rarefied counts and expressed as a percentage. Source data are provided as a Source Data file.

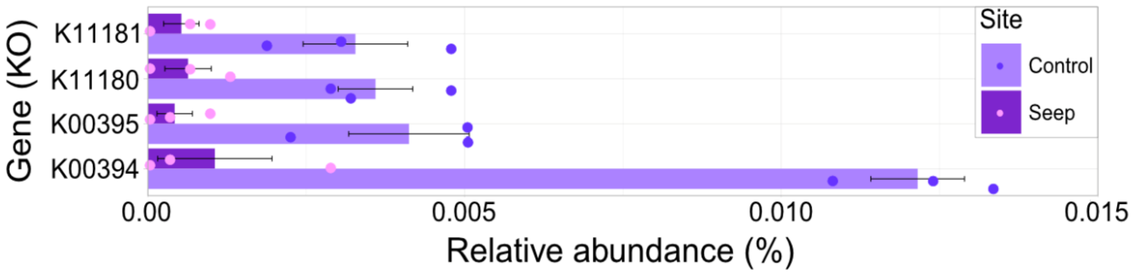

Supplementary Figure 15. Functions involved in dissimilatory sulfide assimilation in *C. singaporensis*. Mean (n = 3 ± s.e.m) relative abundance calculated on rarefied counts and expressed as a percentage. Source data are provided as a Source Data file.

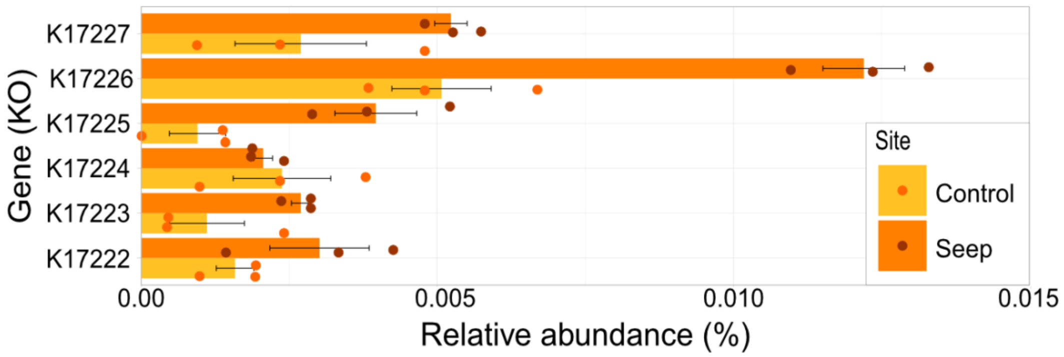

Supplementary Figure 16. Functions involved sulfate oxidation in *S. flabelliformis*. Mean (n = 3 ± s.e.m) relative abundance calculated on rarefied counts and expressed as a percentage. Source data are provided as a Source Data file.

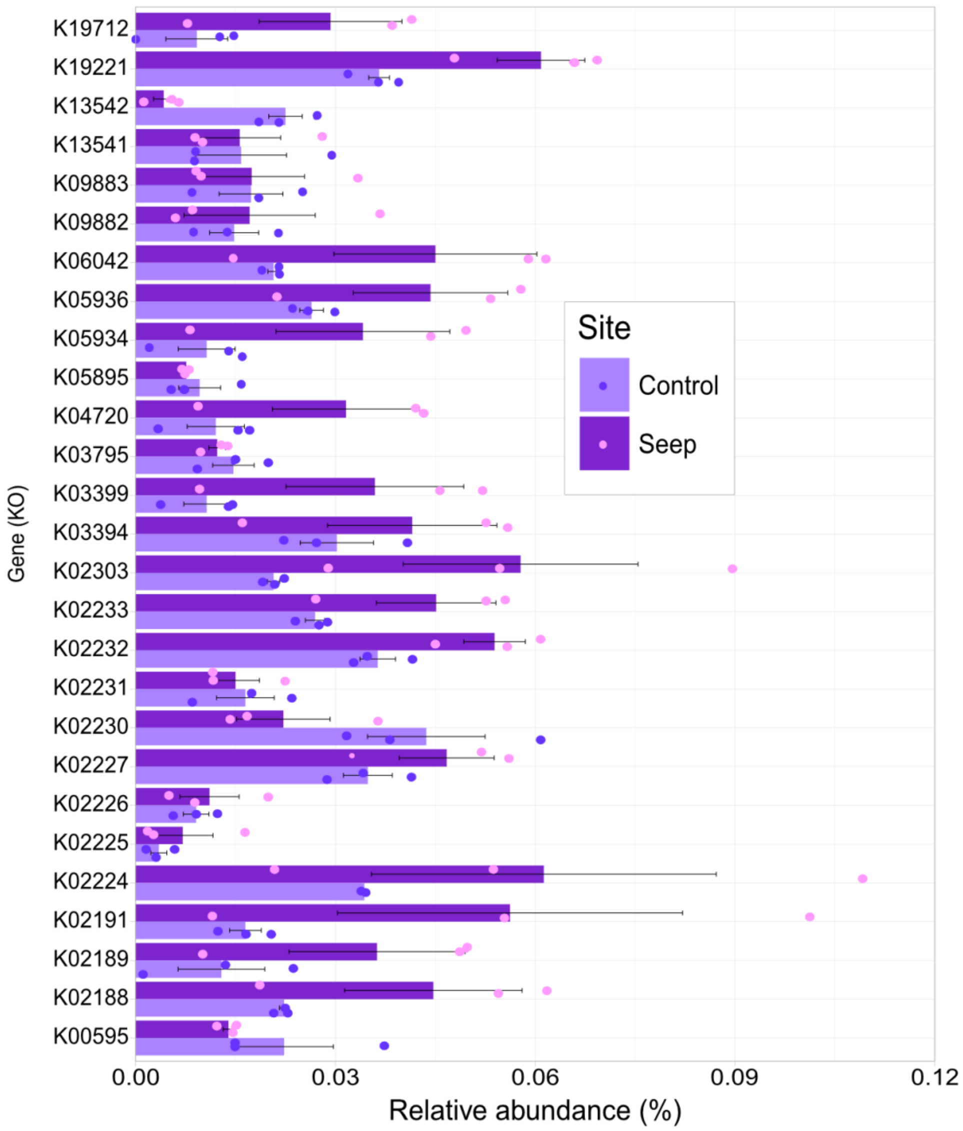

Supplementary Figure 17. Functions involved in cobalamin biosynthesis in *C. singaporensis*. Mean (n = 3 ± s.e.m) relative abundance calculated on rarefied counts and expressed as a percentage. Source data are provided as a Source Data file.

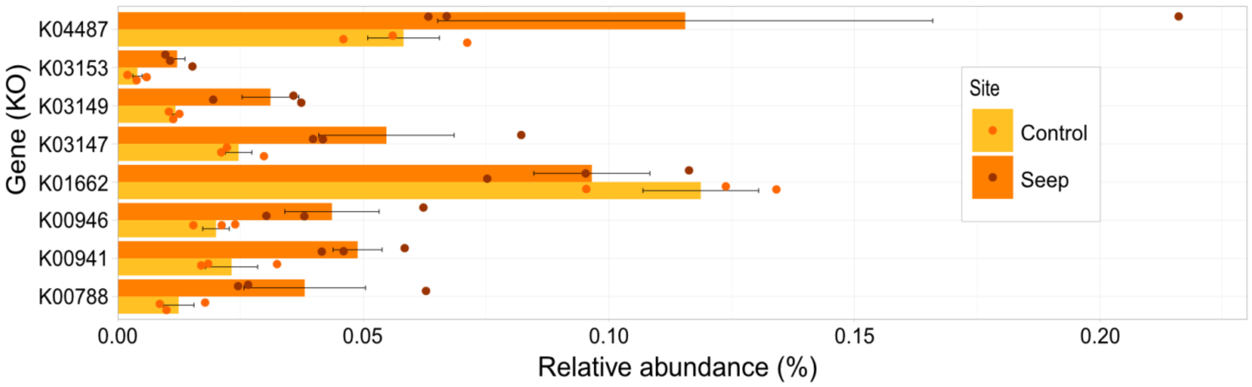

Supplementary Figure 18. Functions involved in thiamin biosynthesis in *S. flabelliformis*. Mean (n = 3 ± s.e.m) relative abundance calculated on rarefied counts and expressed as a percentage. Source data are provided as a Source Data file.

Supplementary Tables

Supplementary Table 1. Number of genes (KOs) significantly enriched at the control or seep site for each of the three sponge species. Values expressed as percentage of total statistically significantly enriched genes (ANOVA) are shown in parentheses.

	C. singaporensis	***S. flabelliformis***	***C. australiensis***
Number of KO enriched at control ($p < 0.05$)	1556 (80%)	369 (20%)	245 (52%)
Number of KO enriched at seep ($p < 0.05$)	379 (20%)	1422 (80%)	230 (48%)

Supplementary Table 2. Statistical output of Permutational Multivariate Analysis of Variance (PERMANOVA) on Bray-Curtis matrix of KOs.

	**Degrees of freedom**	**Sum of Square**	**Mean Square**	**Pseudo-F**	***P* (perm)**	**Unique permutations**
Species	2	4699	2349.5	23.267	0.0001	9940
Site	1	244.18	244.18	2.4181	0.0683	9957
Species x Site	2	548.35	274.18	2.7151	0.0237	9935
Residuals	11	1110.8	100.98			
Total	16	6602.3				

Supplementary Table 3. Summary statistics of environmental variables related to temperature (Temp), salinity, chlorophyll A (Chl a), phaeophytin (Phaeo) and total Nitrogen (TN).

	**Temp (°C)**	**Salinity (ppt)**	**Chl A (µg.L^{-1})**	**Phaeo (µg.L^{-1})**	**TN (µg.L^{-1})**
Avg. C	28.69	35.01	0.243	0.177	14.82
Med. C	29.06	34.80	0.230	0.175	14.48
SD C	1.37	0.63	0.089	0.018	3.72
Min C	26.09	33.20	0.143	0.159	10.30
Max C	31.47	36.00	0.388	0.197	19.90
Avg. S	28.82	34.77	0.190	0.165	15.32
Med. S	29.15	34.80	0.195	0.179	14.93
SD S	1.38	0.54	0.014	0.030	0.94
Min S	26.16	32.20	0.168	0.120	14.37
Max S	31.51	36.00	0.205	0.191	16.73
t-test df	1515.8	162.01	17.996	10.89	13.057
t-test *p*	0.068	1.7e-4	0.247	0.623	0.699
Time range	Mar-14	Apr-14	Jan-13	Jan-13	Jan-13

Avg: Average, Med: Median, , SD: standard deviation, Min: Minimum, Max: Maximum, t-test df: t-test degree of freedom, t-test *p*: t-test *p*-value, C: control, S: seep

Supplementary Table 4. Summary statistics of environmental variables related to carbon: Total Organic Carbon (TOC), pH, Total Aragonite (TA), CO_2 partial pressure (pCO_2), bicarbonate (HCO_3), carbonate (CO_3), carbon dioxide (CO_2) Dissolved Inorganic Carbon (DIC).

	**TOC ($\mu g.L^{-1}$)**	**pH**	**TA ($\mu mol.Kg^{-1}$)**	**pCO_2 (μatm)**	**HCO_3^- ($\mu mol.kg^{-1}$)**	**CO_3^{2-} ($\mu mol.Kg^{-1}$)**	**CO_2 ($\mu mol.kg^{-1}$)**	**DIC ($\mu mol.kg^{-1}$)**
Avg. C	93.62	8.01	2264	369.7	1661	243.0	9.426	1913
Med. C	94.87	8.01	2254	351.7	1660	246.5	9.133	1924
SD C	16.90	0.06	33	55.7	45	24.6	1.380	28
Min C	70.65	7.88	2202	296.5	1574	187.9	7.654	1844
Max C	115.09	8.09	2340	540.3	1761	281.6	13.593	1987
Avg. S	110.08	7.76	2305	827.4	1887	168.8	21.178	2077
Med. S	103.83	7.82	2305	615.6	1857	177.0	15.533	2049
SD S	31.55	0.20	49	713.9	140	44.5	17.940	114
Min S	81.72	6.98	2180	328.4	1679	29.6	8.905	1915
Max S	161.81	8.02	2541	6314.6	2365	249.3	158.077	2552
t-test df	12.618	271.5	155.03	215.27	271.88	198.23	214.99	264.11
t-test *p*	0.246	2.2e-16	5.9e-13	< 2.2e-16	< 2.2e-16	< 2.2e-16	2.2e-16	< 2.2e-16
Time range	Jan-2013	Dec-2011 - Apr-2014	Dec-2011 - Apr-2014	Dec-2011 - Apr-2014	Dec-2011 - Apr-2014	Dec-2011 - Apr-2014	Dec-2011 - Apr-2014	Dec-2011 - Apr-2014

Avg: Average, Med: Median, , SD: standard deviation, Min: Minimum, Max: Maximum, t-test df: t-test degree of freedom, t-test *p*: t-test *p*-value, C: control, S: seep

Supplementary Table 5. Summary statistics of environmental variables related to ions and trace elements: Calcium (Ca), Barrium (Ba), Chloride (Cl), Poatassium (K), Magnesium (Mg), Sodium (Na), Sulfur (S) and Strontium (Sr).

	**Ca ($mg.L^{-1}$)**	**Ba ($mg.L^{-1}$)**	**Cl ($mg.L^{-1}$)**	**K ($mg.L^{-1}$)**	**Mg ($mg.L^{-1}$)**	**Na ($mg.L^{-1}$)**	**S ($mg.L^{-1}$)**	**Sr ($mg.L^{-1}$)**
Avg. C	421.7	0.0018	21739	365.7	1369	10182	902.5	8.60
Med. C	421.0	0.0020	22428	365.0	1367	10151	901	8.58
SD C	3.4	0.0005	2012	1.8	11	79	3.885872	0.06
Min C	419.0	0.0010	18115	364.0	1361	10126	899	8.54
Max C	428.0	0.0020	23943	369.0	1390	10339	910	8.71
Avg. S	420.8	0.0023	21934	364.9	1364	10147	899.9414	8.62
Med. S	421.0	0.0018	21755	364.8	1364	10153	899.6141	8.61
SD S	2.3	0.0019	1654	1.7	7	58	3.193955	0.07
Min S	0.0097	0.0007	18353	361.9	1352	10059	894.721	8.50
Max S	0.0100	0.0106	25693	370.1	1380	10263	907.1617	8.80
t-test df	5.9433	18.974	6.4222	6.9111	5.9972	6.119	6.4222	8.4419
t-test *p*	0.556	0.214	0.830	0.374	0.305	0.350	0.178	0.446
Time range	Aug-2010	Aug-2010	Aug-2010	Aug-2010	Aug-2010	Aug-2010	Aug-2010	Aug-2010

Avg: Average, Med: Median, , SD: standard deviation, Min: Minimum, Max: Maximum, t-test df: t-test degree of freedom, t-test *p*: t-test *p*-value, C: control, S: seep

Supplementary Table 6. Canonical eigenvectors on averages of: carbon dioxide (CO_2), CO_2 partial pressure (pCO_2), carbonate (CO_3^{2-}), Barrium (Ba), chlorophyll A (Chl a), Total Organic Carbon (TOC), bicarbonate (HCO_3^-), Dissolved Inorganic Carbon (DIC), phaeophytin (Phaeo), Total Nitrogen (TN), pH, Total Aragonite (TA), Chloride (Cl), Salinity, Temperature (Temp), Magnesium (Mg), Sodium (Na), Calcium (Ca), Sulfur (S) and Strontium (Sr).

**Variable**	**CAP1**	**CAP2**
Temp (°C)	-0.005	0.322
Salinity (ppt)	0.008	0
Chl a (µg.L^{-1})	0.235	-0.255
Phaeo (µg.L^{-1})	0.075	0.291
TOC (µg.L^{-1})	-0.163	-0.397
TN (µg.L^{-1})	-0.036	0
pH	0.034	0.191
TA (µmoles.Kg^{-1})	-0.02	-0.05
pCO2 (µatm)	-0.594	-0.349
HCO3 (µmol.kg^{-1})	-0.129	0.041
CO_3^{2-} (µmol.kg^{-1})	0.327	-0.644
CO_2 (µmol.kg^{-1})	-0.596	0.011
DIC (µmol.kg^{-1})	-0.086	-0.062
Ba (mg.L^{-1})	-0.266	0.085
Cl (mg.L^{-1})	-0.01	0.017
Mg (mg.L^{-1})	0.004	0.011
Na (mg.L^{-1})	0.004	0.007
S (mg.L^{-1})	0.003	0.009
Sr (mg.L^{-1})	-0.003	-0.003
Ca (mg.L^{-1})	0.002	0.007
K (mg.L^{-1})	0.002	0.006

Supplementary Table 7. Taxonomy of scaffolds carrying genes encoding HP/HB cycle enzymes, as a percentage of the total number of such scaffolds, for each gene.

	**Gene**	**Major taxa**	**Average at control (%) ± SD**	**Average at seep (%) ± SD**
**K18603**	Acetyl-CoA/propionyl-CoA carboxylase [EC:6.4.1.2 6.4.1.3]	*Nitrosopumilus*	100	100
**K18604**	Acetyl-CoA/propionyl-CoA carboxylase [EC:6.4.1.2 6.4.1.3]	*Nitrosopumilus*	80 (± 17)	96 (± 4)
**K18605**	Acetyl-CoA/propionyl-CoA carboxylase [EC:6.4.1.2 6.4.1.3]	*Nitrosopumilus*	100	75 (± 15)
**K18602**	Malonic semialdehyde reductase [EC:1.1.1.-]	*Nitrosopumilus*	75 (± 43), 100% Thaumarchaeota	95 (± 8)
**K18594**	3-hydroxypropionyl-CoA synthetase (ADP-forming) [EC:6.2.1.-]	*Nitrosopumilus maritimus*	79 (± 11), 90% Thaumarchaeota	98 (± 2)
K01715	Crotonase, enoyl-CoA hydratase	Bacteria and unclassified	100	97 (± 3)
**K15019**	3-hydroxypropionyl-coenzyme A dehydratase [EC:4.2.1.116] and crotonly-CoA hydratase	*Nitrosopumilus*	72 (± 13)	100
K19745	Acryloyl-CoA reductase	Bacteria and unclassified	100	100
K15052	Propionyl-CoA carboxylase [EC:6.4.1.3]	Bacteria and unclassified	100	100
**K05606**	Methylmalonyl-CoA epimerase	Thaumarchaeota	20 (± 16)	58 (± 33)
**K01848**	Methylmalonyl-CoA mutase [EC:5.4.99.2] (MUT)	Thaumarchaeota	37 (± 28)	79 (± 1)
**K01849**	Methylmalonyl-CoA mutase [EC:5.4.99.2] (MUT)	Thaumarchaeota	48 (± 31)	63 (± 52)
**K18593**	4-hydroxybutyryl-CoA synthetase (ADP-forming) [EC:6.2.1.-]	*Nitrosopumilus*	97 (± 5)	100 (± 1)
**K14534**	4-hydroxybutyryl-CoA dehydratase / vinylacetyl-CoA-Delta-isomerase [EC:4.2.1.120 5.3.3.3]	*Nitrosopumilus*	79 (± 11)	99 (± 1)
**K15016**	3-hydroxybutyryl-CoA dehydrogenase [EC:4.2.1.17 1.1.1.35]	*Nitrosopumilus*	71 (± 20)	69 (± 14)
**K00626**	Acetyl-CoA acetyltransferase	Bacteria and Archaea	70.1 (± 5) and 11.9 (± 7)	48 (± 17) and 38.5 (± 24)

Bold indicates KOs used in Figure3.

Supplementary Table 8. Taxonomy of *C. singaporensis* scaffolds carrying genes for creatine, creatinine and arginine catabolism, and ammonium utilisation as a percentage of the total number of such scaffolds, for each gene.

	Gene	Average bacteria in control (%)	Average bacteria in seep (%)	Average archaea in control (%)	Average archaea in seep (%)
K01485	Cytosine deaminase	81 (± 4)	91 (± 9)	0	0
K01473	N-methylhydantoinase (hyuA)	79 (± 10)	73 (± 19)	0	0
K01474	N-methylhydantoinase (hyuB)	79 (± 8)	71 (± 11)	1 (± 1)	0
K08687	N-carbamoylsarcosine amidase	83 (± 29)	0	0	0
K08688	Creatinase	78 (± 20)	70 (± 39)	0	0
K01430	UreA (Urease subunit)	34 (± 32)	15 (± 6)	62 (± 39)	85 (± 6)
K01429	UreB (Urease subunit)	43 (± 39)	15 (± 3)	57 (± 39)	84 (± 4)
K01428	UreC (Urease subunit)	49 (± 31)	21 (± 11)	47 (± 33)	78 (± 12)
K03190	UreD-UreH (Urease subunit)	38 (± 27)	16 (± 5)	53 (± 24)	84 (± 5)
K03187	UreE (Urease subunit)	36 (± 32)	13 (± 1)	60 (± 40)	86 (± 1)
K03188	UreF (Urease subunit)	42 (± 34)	14 (± 3)	56 (± 35)	86 (± 3)
K03189	UreG (Urease subunit)	43 (± 35)	13 (± 2)	55 (± 34)	87 (± 2)
K03192	UreJ (Urease subunit)	97 (± 8)	100	0	0
K03191	UreI (Urea transport)	67 (± 57)	100	0	0
K11959	UrtA (Urea transport)	97 (± 2)	81 (± 32)	0	0
K11960	UrtB (Urea transport)	99 (± 2)	97 (± 5)	0	0
K11961	UrtC (Urea transport)	92 (± 7)	83 (± 29)	0	0
K11962	UrtD (Urea transport)	85 (± 13)	85 (± 25)	0	0
K11963	UrtE (Urea transport)	82 (± 15)	93 (± 12)	0	0
K01480	Agmatinase	62 (± 18)	48 (± 15)	26 (± 16)	41 (± 20)
K01585	Arginine decarboxylase	95 (± 9)	94 (± 11)	0	0
K02626	Arginine decarboxylase	11 (± 6)	4 (± 4)	62 (± 38)	98 (± 3)
K01915	Glutamine synthetase	75 (± 2)	57 (± 8)	12 (± 8)	30 (± 18)
K10944	AmoA	13 (± 18)	0 (± 1)	87 (± 18)	100 (± 1)
K10945	AmoB	7 (± 6)	0	74 (± 23)	100
K10946	AmoC	9 (± 4)	0	75 (± 20)	100

Supplementary Table 9. Taxonomy of *C. singaporensis* scaffolds carrying genes for arginine biosynthesis as a percentage of the total number of such scaffolds, for each gene.

	Gene	Average bacteria in control (%)	Average bacteria in seep (%)	Average archaea in control (%)	Average archaea in seep (%)
K00619	*argA*	35 (± 11)	3 (± 4)	63 (± 10)	96 (± 3)
K14682	*argAB*	93 (± 9)	82 (± 15)	0	0
K00620	*argJ*	86 (± 8)	78 (± 5)	0	0
K00930	*argB*	87 (± 12)	89 (± 7)	0	0
K00145	*argC*	81 (± 7)	82 (± 4)	0	0
K00818	*argD*	82 (± 27)	90 (± 12)	0	0
K00821	*argD*	83 (± 22)	80 (± 16)	0	0
K01438	*argE*	82 (± 14)	71 (± 24)	0	1
K05826	*LysW*	26 (± 16)	6 (± 9)	74 (± 16)	94 (± 9)
K05827	*LysX-argX*	35 (± 25)	3 (± 2)	58 (± 30)	96 (± 1)
K05828	*LysZ*	31 (± 25)	0	68 (± 25)	100
K05829	*LysY*	44 (± 28)	3 (± 1)	54 (± 25)	96 (± 1)
K05830	*LysJ*	41 (± 30)	1 (± 1)	52 (± 28)	99 (± 1)
K05831	*LysK*	22 (± 19)	2 (± 2)	75 (± 16)	96 (± 2)

Supplementary Table 10. Key features of the *lysX/argX* gene (K05827) from *C. singaporensis* samples.

Sample	Site	Scaffold ID	Gene ID	Identity of residues 259-260 on lysX/argX	Species (IMG-assigned)
co6ic	Control	3300021558 assembled Ga0224704_1093963	Ga0224704_10939635	Gly-Phe	*Candidatus Nitrosopumilus sediminis*
co6ic	Control	3300021558 assembled Ga0224704_1126348	Ga0224704_112634814	Gly-Phe	*Nitrosopumilus maritimus*
co6ic	Control	3300021558 assembled Ga0224704_1156405	Ga0224704_11564052	Asn-Thr	*Nitrosopumilus maritimus*
co14ic	Control	3300021557 assembled Ga0224710_1181710	Ga0224710_11817102	Asn-Thr	*Nitrosopumilus maritimus*
co15ic	Control	3300021552 assembled Ga0224709_1066020	Ga0224709_10660203	Asn-Thr	*Nitrosopumilus maritimus*
co15ic	Control	3300021552 assembled Ga0224709_1088276	Ga0224709_10882767	Gly-Lys	*Candidatus Nitrosopumilus sediminis*
co15ic	Control	3300021552 assembled Ga0224709_1089456	Ga0224709_108945611	Gly-Lys	*Nitrosopumilus maritimus*
co15ic	Control	3300021552 assembled Ga0224709_1089456	Ga0224709_108945618	Asn-Thr	*Nitrosopumilus maritimus*
co35is	Seep	3300021549 assembled Ga0224705_1076848	Ga0224705_107684811	Gly-Lys	*Nitrosopumilus maritimus*
co35is	Seep	3300021549 assembled Ga0224705_1076848	Ga0224705_107684818	Asn-Thr	*Nitrosopumilus maritimus*
co35is	Seep	3300021549 assembled Ga0224705_1096918	Ga0224705_10969181	Gly-Lys	*Nitrosopumilus species*
co46is	Seep	3300021545 assembled Ga0224706_1081283	Ga0224706_108128310	Gly-Lys	*Nitrosopumilus maritimus*
co46is	Seep	3300021545 assembled Ga0224706_1081283	Ga0224706_108128317	Asn-Thr	*Nitrosopumilus maritimus*
co52is	Seep	3300021544 assembled Ga0224707_111780	Ga0224707_11178010	Asn-Thr	*Nitrosopumilus maritimus*
co52is	Seep	3300021544 assembled Ga0224707_111780	Ga0224707_11178017	Gly-Lys	*Nitrosopumilus maritimus*

Supplementary Table 11. Taxonomy of *S. flabelliformis* scaffolds carrying genes for creatine and creatinine catabolism and ammonium utilisation, as a percentage of the total number of such scaffolds, for each gene.

	Gene	Average bacteria in control (%)	Average bacteria in seep (%)	Average archaea in control (%)	Average archaea in seep (%)
K01485	Cytosine deaminase	100	92 (± 14)	0	0
K01473	N-methylhydantoinase (hyuA)	92 (± 7)	85 (± 9)	3 (± 5)	3 (± 5)
K01474	N-methylhydantoinase (hyuB)	98 (± 4)	96 (± 3)	0	0
K08688	Creatinase	94 (± 9)	76 (± 14)	0	0
K01430	UreA (Urea transport)	100	89 (± 16)	0	0
K01429	UreB (Urea transport)	80 (± 34)	75 (± 15)	0	0
K01428	UreC (Urea transport)	90 (± 11)	92 (± 9)	0	0
K03190	UreD-UreH (Urea transport)	89 (± 7)	55 (± 8)	0	1 (± 2)
K03187	UreE (Urea transport)	100	99 (± 1)	0	0
K03188	UreF (Urea transport)	98 (± 2)	90 (± 9)	0	0
K03189	UreG (Urea transport)	83 (± 27)	84 (± 14)	0	0
K03192	UreJ (Urea transport)	100	100	0	0
K01915	Glutamine synthetase	87 (± 2)	80 (± 15)	4 (± 4)	12 (± 16)
K00265	Glutamate synthase	99 (± 1)	87 (± 9)	0	0
K00266	Glutamate synthase	79 (± 6)	89 (± 3)	0	0

Supplementary Table 12. Taxonomy of *S. flabelliformis* scaffolds carrying genes for taurine degradation, sulfide production and assimilation, as a percentage of the total number of such scaffolds, for each gene.

	Gene	Major taxa	Average at control (%)	Average at seep (%)
K03851	Taurine pyruvate aminoitransferase	Thiotrichales	41% (± 6)	50 (± 16)
K03852	Sulfoacetaldehyde acetyltransferase	Burkholderiales	33 (± 4)	10 (± 8)
K00381	Sulfite reductase	Methylcoccales	35 (± 9)	9.5 (± 8.3)
K00651	Homoserine O-succinyl-transferase	Chromatiales<br>Methylococcales	31.8 (± 8.3)<br>38.9 (± 14.2)	3.8 (± 6.4)<br>0
K01739	Cystathionine gamma-synthase	Myxococcales	73 (+- 4)	47 (+- 8)
K01760	Cystathionine-β-lyase	Myxococcales	78 (± 8)	48 (± 34)
K10764	O-succinyl-homoserine sulfydrylase	Oceanospirillales	28.8 (± 14)	6.2 (± 7)

Supplementary Table 13. Taxonomy of *C. singaporensis* scaffolds carrying genes involved in sulfate reduction as percentage of the total number of such scaffolds, for each gene.

	Gene	Major taxa	Average at control (%)	Average at seep (%)
K00394	*aprA*	Archaeoglobales<br>Chromatiales<br>Gammaproteobacteria	6<br>4<br>10	75<br>25<br>0
K00395	*aprB*	Chromatiales<br>Gammaproteobacteria	50<br>23	100<br>0
K11180	*dsrA*	Chromatiales	70 (± 53)<br>(100% Proteobacteria)	44 (± 51),<br>Absent in one sample
K11181	*dsrB*	Gammaproteobacteria	81 (± 16)	67 (± 58)<br>Absent in one sample

Supplementary Table 14. Taxonomy of *S. flabelliformis* scaffolds carrying genes involved in sulfate reduction as percentage of the total number of such scaffolds, for each gene.

	Gene	Major taxa	Average at control (%)	Average at seep (%)
K17222	soxA	Gammaproteobacteria	66.7 (± 57.7)	100
K17223	soxX	Gammaproteobacteria and/or unclassified	90 (± 17.3)	100
K17224	soxB	Gammaproteobacteria	88 (± 22.4)	100
K17225	*soxC*	Gammaproteobacteria and Alphaproteobacteria	100	100
K17226	*soxY*	Gammaproteobacteria	65 (± 11)	63 (± 17)
K17227	*soxZ*	Gammaproteobacteria and/or unclassified	100	100

Supplementary Table 15. Results of ANOVA testing the effects of site and oxygen condition on the relative abundance of genes involved in the synthesis of cobalamin in the *C. singaporensis* microbiome.

	Df	Sum of Squares	Mean Square	F value	P-value
Site	1	0.21451	0.21451	6.897	0.0304
Oxygen condition	1	0.02824	0.02824	0.908	0.3685
Site x Oxygen condition	1	0.01791	0.01791	0.576	0.4697
Residuals	8	0.24881	0.03110		

Supplementary Table 16. Taxonomy of scaffolds carrying genes for cobalamin biosynthesis, as a percentage of the total number of such scaffolds, for each type of pathway.

Pathway	Major phyla	Average at control (%) ± stdev	Average at seep (%)
Aerobic	Cyanobacteria<br>Proteobacteria<br>Thaumarchaeota	8 (± 7)<br>13 (± 10)<br>19 (± 15)	7 (± 6)<br>14 (± 19)<br>26 (± 15)
Anaerobic	Cyanobacteria<br>Proteobacteria<br>Thaumarchaeota	8 (± 6)<br>10 (± 9)<br>19 (± 15)	6 (± 6)<br>9 (± 15)<br>26 (± 16)

Supplementary Table 17. Taxonomy of scaffolds carrying the genes for thiamin biosynthesis with differences between sites, expressed as a percentage of the total number of such scaffolds, for each gene.

	Gene	Major taxa	Average at control (%)	Average at seep (%)
K03147	*ThiC*	Proteobacteria<br>Thaumarchaeota	19 (± 4)<br>39 (± 13)	15 (± 10)<br>31 (± 37)
K00941	*ThiD*	Proteobacteria<br>Thaumarchaeota	36 (± 18)<br>51 (± 10)	57 (± 25)<br>29 (± 32)
K00788	*ThIE*	Proteobacteria	50 (± 12)	37 (± 15)
K00946	*ThiL*	Proteobacteria<br>Thaumarchaeota	44 (± 24)<br>41 (± 18)	65 (± 30)<br>27 (± 31)
K03153	*ThiO*	Cyanobacteria<br>Proteobacteria	21 (± 13)<br>79 (± 13)	14 (± 1)<br>90 (± 9)
K03149	*ThiG*	Proteobacteria	68 (± 30)	60 (± 16)
K01662	*Dxs*	Proteobacteria	85 (± 5)	69 (± 12)
K04487	*IscS*	Actinobacteria<br>Proteobacteria<br>Thaumarchaeota	18 (± 7)<br>21 (± 15)<br>14 (± 5)	7 (± 11)<br>21 (± 12)<br>7 (± 6)

Supplementary Table 18. Statistics of sequence processing.

Sample ID	Species and Site	Number of raw reads	Number of reads after quality trimming	Input mean length (bp)	Number of contigs after assembly	% of Eukaryotic contamination (removed)	% of reads mapped to assembly
co6ic	*C. singaporensis*, Control	4886130	4872020	242.51	192726	3.2	75.76
co14ic	*C. singaporensis*, Control	6711024	6699546	238	328680	5.0	73.91
co15ic	*C. singaporensis*, Control	4203192	4191894	248.77	163960	1.6	76.33
co35is	*C. singaporensis*, Seep	4093436	4085846	256.22	191919	1.5	67.07
co46is	*C. singaporensis*, Seep	4440394	4431782	253.02	165209	2.5	71.33
co52is	*C. singaporensis*, Seep	3835628	3824370	243.33	136182	2.1	71.47
st9ic	*S. flabelliformis*, Control	6369926	6348546	243.53	190377	4.1	66.71
st32ic	*S. flabelliformis*, Control	5316804	5299250	239.7	177493	5.3	65.16
st36ic	*S. flabelliformis*, Control	7209704	7185022	239.11	281247	8.0	67.72
st7is	*S. flabelliformis*, Seep	5415092	5392310	236.96	274471	8.8	61.76
st20is	*S. flabelliformis*, Seep	6460136	6438298	243.88	284012	8.8	67.91
st43is	*S. flabelliformis*, Seep	5693478	5675070	245.75	255794	8.4	63
cg10ic	*C. australiensis*, Control	5542238	5530192	224.32	151120	6.1	67.87
cg14ic	*C. australiensis*, Control	4170450	4160532	249.81	100913	2.8	73.18
cg17ic	*C. australiensis*, Control	3467246	3454020	238.74	118017	6.6	58.39
cg23is	*C. australiensis*, Seep	3278348	3264828	228.72	127317	7.0	53.33
cg24is	*C. australiensis*, Seep	5115336	5089038	229.13	132650	5.7	63.42
cg27is	*C. australiensis*, Seep	5490684	5456424	211.18	199402	8.9	55.06
EBic1	Water, Control	5169462	5153944	244.2	243718	0	58.62
wic	Water, Control	2482988	2479080	254.37	93724	0	45.14
EBis3	Water, Seep	6020196	6006742	259.05	318552	0	57.03
EBis4	Water, Seep	8719600	8693154	250.5	441685	0	63.19
wis	Water, Seep	5783296	5769448	267.69	332914	0	55.60

Supplementary Table 19. Accession numbers of data available from NCBI's SRA and IMG/MER.

Sample ID	Species and Site	NCBI SRA Accession (study)	NCBI SRA Bioproject	NCBI SRA Accession (sample)	IMG/MER ID
co6ic	*C. singaporensis*, Control	SRP159543	PRJNA488959	SAMN09946148	3300021558
co14ic	*C. singaporensis*, Control	SRP159543	PRJNA488959	SAMN09946149	3300021557
co15ic	*C. singaporensis*, Control	SRP159543	PRJNA488959	SAMN09946150	3300021552
co35is	*C. singaporensis*, Seep	SRP159543	PRJNA488959	SAMN09946151	3300021549
co46is	*C. singaporensis*, Seep	SRP159543	PRJNA488959	SAMN09946152	3300021545
co52is	*C. singaporensis*, Seep	SRP159543	PRJNA488959	SAMN09946153	3300021544
st9ic	*S. flabelliformis*, Control	SRP159543	PRJNA488959	SAMN09946160	3300021550
st32ic	*S. flabelliformis*, Control	SRP159543	PRJNA488959	SAMN09946161	3300021539
st36ic	*S. flabelliformis*, Control	SRP159543	PRJNA488959	SAMN09946162	3300021556
st7is	*S. flabelliformis*, Seep	SRP159543	PRJNA488959	SAMN09946165	3300021554
st20is	*S. flabelliformis*, Seep	SRP159543	PRJNA488959	SAMN09946163	3300021555
st43is	*S. flabelliformis*, Seep	SRP159543	PRJNA488959	SAMN09946164	3300021551
cg10ic	*C. australiensis*, Control	SRP159543	PRJNA488959	SAMN09946154	3300021548
cg14ic	*C. australiensis*, Control	SRP159543	PRJNA488959	SAMN09946155	3300021543
cg17ic	*C. australiensis*, Control	SRP159543	PRJNA488959	SAMN09946156	3300021542
cg23is	*C. australiensis*, Seep	SRP159543	PRJNA488959	SAMN09946157	3300021547
cg24is	*C. australiensis*, Seep	SRP159543	PRJNA488959	SAMN09946158	3300021546
cg27is	*C. australiensis*, Seep	SRP159543	PRJNA488959	SAMN09946159	3300021553
EBic1	Water, Control	SRP159543	PRJNA488959	SRR7783602	3300007144
wic	Water, Control	SRP159543	PRJNA488959	SRR7783603	3300007041
EBis3	Water, Seep	SRP159543	PRJNA488959	SRR7783604	3300007116
EBis4	Water, Seep	SRP159543	PRJNA488959	SRR7783601	3300007114
wis	Water, Seep	SRP159543	PRJNA488959	SRR7783605	3300007113

Supplementary Table 20. Origin and properties of MAGs from *C. singaporensis* and *S. flabelliformis*.

Host	Site	Bin ID	% Compl.	% Contam.	Quality	GTDB taxonomy
C. singaporensis	C	metabat_ coeloUpaCtrlOnly.1	95.44	3.6	77.44	d_Bacteria;p_Latescibacteria;c_UBA2968
C. singaporensis	C	metabat_ coeloUpaCtrlOnly.10	73.03	1.35	66.28	d_Bacteria;p_Proteobacteria;c_Gammaproteobacteria;o_UBA11654;f_UBA11654
C. singaporensis	C	metabat_ coeloUpaCtrlOnly.25	91.6	1.68	83.2	d_Bacteria;p_Dadabacteria;c_UBA1144
C. singaporensis	C	metabat_ coeloUpaCtrlOnly.3	89.85	5.49	62.4	d_Bacteria;p_Poribacteria;c_WGA-4E;o_WGA-4E;f_WGA-3G;g_WGA-3G
C. singaporensis	C	metabatSS_ coeloUpaCtrlOnly.13	75.43	1.47	68.08	d_Bacteria;p_Proteobacteria;c_Gammaproteobacteria;o_Pseudomonadales;f_Pseudohongiellaceae
C. singaporensis	C	metabatSS_ coeloUpaCtrlOnly.2	95.44	5.49	67.99	d_Bacteria;p_Poribacteria;c_WGA-4E;o_WGA-4E;f_WGA-3G;g_WGA-3G
C. singaporensis	C	metabatSS_ coeloUpaCtrlOnly.26	77.02	0.07	76.67	d_Archaea;p_Crenarchaeota;c_Nitrososphaeria;o_Nitrososphaerales;f_Nitrosopumilaceae
C. singaporensis	C	metabatSS_ coeloUpaCtrlOnly.7	87.91	0.99	82.96	d_Bacteria;p_Chloroflexi;c_Dehalococcoidia
C. singaporensis	C	metabatVS_ coeloUpaCtrlOnly.15	93.34	0.68	89.94	d_Bacteria;p_Cyanobacteria;c_Oxyphotobacteria;o_Synechococcales_A;f_Cyanobiaceae;g_Synechococcus_B
C. singaporensis	C	metabatVS_ coeloUpaCtrlOnly.18	78.48	1.24	72.28	d_Bacteria;p_Proteobacteria;c_Alphaproteobacteria;o_Caulobacterales;f_Maricaulaceae;g_Oceanicaulis
C. singaporensis	C	metabatVS_ coeloUpaCtrlOnly.21	91.35	0.97	86.5	d_Archaea;p_Crenarchaeota;c_Nitrososphaeria;o_Nitrososphaerales;f_Nitrosopumilaceae;g_Nitrosopumilus
C. singaporensis	C	metabatVS_ coeloUpaCtrlOnly.27	71.13	0.9	66.63	d_Bacteria;p_Proteobacteria;c_Gammaproteobacteria;o_UBA4575;f_UBA4575
C. singaporensis	C	metabatVS_ coeloUpaCtrlOnly.8	67.32	1.43	60.17	d_Bacteria;p_Proteobacteria;c_Alphaproteobacteria;o_Rhodobacterales;f_Rhodobacteraceae;g_Albidovulum
C. singaporensis	S	metabat_ coeloUpaSeepOnly.2	77.97	0.83	73.82	d_Bacteria;p_Proteobacteria;c_Gammaproteobacteria;o_Pseudomonadales;f_Pseudohongiellaceae
C. singaporensis	S	metabatSS_ coeloUpaSeepOnly.3	77.76	0.27	76.41	d_Bacteria;p_Proteobacteria;c_Gammaproteobacteria;o_Pseudomonadales;f_HTCC2089
C. singaporensis	S	metabatSS_ coeloUpaSeepOnly.4	92.26	0.36	90.46	d_Bacteria;p_Cyanobacteria;c_Oxyphotobacteria;o_Synechococcales_A;f_Cyanobiaceae;g_Synechococcus_B
C. singaporensis	S	metabatSS_ coeloUpaSeepOnly.9	68.94	0.42	66.84	d_Bacteria;p_Dadabacteria;c_UBA1144
C. singaporensis	S	metabatVS_ coeloUpaSeepOnly.5	69.08	0.52	66.48	d_Bacteria;p_Proteobacteria;c_Alphaproteobacteria;o_Caulobacterales;f_Maricaulaceae;g_Oceanicaulis
C. singaporensis	S	metabatVS_ coeloUpaSeepOnly.9	68.61	0.97	63.76	d_Archaea;p_Crenarchaeota;c_Nitrososphaeria;o_Nitrososphaerales;f_Nitrosopumilaceae;g_Nitrosopumilus
S. flabelliformis	C	metabatSS_ styUpaCtrlOnly.2	83.96	0.91	79.41	d_Bacteria;p_Nitrospirota;c_Nitrospiria;o_Nitrospirales;f_UBA8639
S. flabelliformis	C	metabatSS_ styUpaCtrlOnly.3	85.01	0.61	81.96	d_Bacteria;p_Proteobacteria;c_Gammaproteobacteria;o_UBA10353
S. flabelliformis	S	metabatSS_ styUpaSeepOnly.2	73.97	0.35	72.22	d_Bacteria;p_Proteobacteria;c_Gammaproteobacteria;o_UBA4575;f_UBA4575
S. flabelliformis	S	metabatSS_ styUpaSeepOnly.3	82.28	1.22	76.18	d_Bacteria;p_Proteobacteria;c_Gammaproteobacteria;o_UBA10353;f_UBA3123
S. flabelliformis	S	metabatVS_ styUpaSeepOnly.4	75.26	0	75.26	d_Archaea;p_Crenarchaeota;c_Nitrososphaeria;o_Nitrososphaerales;f_Nitrosopumilaceae;g_Cenarchaeum

C: control site; S: seep site; Compl.: Completeness; Contam,: Contamination.

Supplementary methods

3-hydroxypropionate/4-hydroxybutyrate cycle

KO terms associated with the 3-hydroxyproprionate/4-hydroxybutyrate cycle were carefully chosen to produce panel a of Figure 3. The vast majority of sponge symbiont genes involved in the HP/HB cycle were assigned to thaumarchaeal scaffolds. Bacterial genes for which no archaeal homologs have yet been identified were generally missing from the metagenomes. The HP/HB cycle in **Figure3** therefore represents KO terms which have been catalogued for Archaea according to gene names, substrates and products as per Konneke et al.[1] and Qin et al.[2] and reflected reactions taking place only in Archaea. The only exception was acetyl-CoA acetyltransferase (K00626), which is present in multiple biochemical pathways so does not contribute exclusively to the HP/HB cycle. The archaeal HP/HB cycle is a topic of current research, hence the following section defines how KOs were selected for construction of **Figure3**, whilst **Supplementary Figure 8** represents relative abundance of KOs used in **Figure3**, and **Supplementary Table 7** contains all KO terms associated with functions performed by HP/HB enzymes (both bacterial and archaeal).

Acetyl-CoA/propionyl-CoA carboxylase (Nmar_0272 and Nmar_0274)

According to Qin et al.[2], *Nmar_0272*, which encodes acetyl-CoA/propionyl-CoA carboxylase (EC number 6.4.1.2, 6.4.1.3), does not correspond to any KO, whilst and *Nmar_0274*, encoding the same gene, corresponds to K18605. Konneke et al.[1] state that *Nmar_0272*, *Nmar_0273* and *Nmar_0274* correspond to both "acetyl-CoA carboxylase" and "propionyl-CoA carboxylase". In the KEGG database the "acetyl-CoA/propionyl-CoA carboxylase" function in the HP/HB cycle is represented by the 3 KO terms K18603, K18604 and K18605. All three KOs were included in the current analysis. K15052, which represents a "propionyl-CoA carboxylase" derived from Bacteria was not represented in **Figure3**.

Malonyl-CoA reductase (unknown in Archaea)

Malonyl-CoA reductase is assigned by the KEGG database to K15017 and K14468, neither of which were detected in any of the *C. singaporensis* samples.

Malonic semialdehyde reductase (Nmar_1110)

According to Qin et al.[2] (Figure3), *Nmar_1110* represents "malonic semialdehyde reductase", yet the SOM of the same publication lists *Nmar_1110* with a KEGG annotation as "K18602, succinate semialdehyde reductase [EC:1.1.1.-]". This is likely to be human error as the KEGG database lists K18602 as "malonic semialdehyde reductase". In addition, *Nmar_1110* was biochemically characterized in *Nitrosopumilus maritimus* and the product found to be highly specific to malonic semialdehyde (Otte et al., 2015). Therefore in **Figure3** of the present manuscript, K18602 represents malonic semialdehyde reductase, whilst the archaeal homolog of succinic semialdehyde reductase remains unknown.

3-hydroxypropionyl-CoA synthetase (ADP-forming) (Nmar_1309)

According to Qin et al.[2], *Nmar_1309* is listed as "K18594, 3-hydroxypropionyl-CoA synthetase (ADP-forming) [EC:6.2.1.-]", which is also consistent with Table 1 of Konneke et al.[1]. The relative abundance of this KO term is therefore represented in **Figure3** as 3-hydroxypropionyl-CoA synthetase.

3-hydroxypropionyl-CoA dehydratase and crotonyl-CoA hydratase (Nmar_1308)

According to the Qin et al.[2] SOM dataset, Nmar_1308 is considered "K01715, 3-hydroxybutyryl-CoA dehydratase (Crotonase) (3-HP/4-HB Cycle enzyme)" but is represented as both 3-hydroxypropionyl-CoA dehydratase and Crotonyl-CoA hydratase on Figure1 of the same paper. In Table 1 of the Konneke et al.[1] publication, *Nmar_1308* corresponds to 3-hydroxypropionyl-CoA dehydratase and crotonyl-CoA hydratase (3-hydroxybutyryl-CoA forming). Further interrogation of the KEGG database showed that this enzyme could also correspond to K15019. Considering the origin of K01715 (exclusively bacterial or unclassified) and K15019 (mostly if not exclusively Archaeal), both 3-hydroxypropionyl-CoA dehydratase and cronoyl-CoA hydratase functions were represented by the relative abundance of K15019 on **Figure3** of the present manuscript.

Acryloyl-CoA reductase (unknown in Archaea)

Acryloyl-CoA reductase is assigned by the KEGG database to K19745, which was detected in all *C. singaporensis* samples (see **Supplementary Table 7** and Source data file).

Methylmalonyl-CoA epimerase and methylmalonyl-CoA mutase (Nmar_0953, Nmar_0954, Nmar_0958)

Methylmalonyl-CoA epimerase and methylmalonyl-CoA mutase were consistently represented by three KOs. Qin et al.[2] and Konneke et al.[1] both reported that *Nmar_0953* corresponds to K05606 (methylmalonyl-CoA/ethylmalonyl-CoA epimerase [EC:5.1.99.1]), *Nmar_0954* corresponds to K01848 (methylmalonyl-CoA mutase, N-terminal domain [EC:5.4.99.2]) and *Nmar_0958* corresponds to K01849 (methylmalonyl-CoA mutase, C-terminal domain [EC:5.4.99.2]). These 3 KO terms were therefore used for Methylmalonyl-CoA epimerase and methylmalonyl-CoA mutase in **Figure3**.

Succinyl-CoA reductase (unknown in Archaea)

Succinyl-CoA reductase is assigned to K15038 in the KEGG database, but was absent from all *C. singaporensis* samples.

Succinic semialdehyde reductase (unknown in Archaea)

Succinic semialdehyde reductase is assigned to K14465 in the KEGG database, but was found in only one sample from each site and therefore not investigated further.

4-hydroxybutyryl-CoA synthetase (ADP-forming) (Nmar_0206)

According to Qin et al.[2], *Nmar_0206* corresponds to a CoA-binding domain protein (3-HP/4-HB Cycle enzyme), with a "K09181, yfiQ; acetyltransferase" description in the KEGG database. However, *Nmar_0206* is listed as a 4-hydroxybutyryl-CoA synthetase (ADP-forming) in Konneke et al.[1]. Likewise, the KEGG database contains a 4-hydroxybutyryl-CoA synthetase (ADP-forming) entry linked to K18593, and we therefore represent this KO on **Figure3**.

4-hydroxybutyryl-CoA dehydratase (Nmar_0207)

Nmar_0207 is listed as a 4-hydroxybutyryl-CoA dehydratase in both Qin et al.[2] and Konneke et al.[1] and the corresponding KO, K14534, is therefore represented in **Figure3**.

3-hydroxybutyryl-CoA dehydrogenase (Nmar_1028)

Nmar_1028 corresponds to K15016, 3-hydroxybutyryl-CoA dehydrogenase in both Qin et al.[2] and Konneke et al.[1], and this KO is therefore represented in **Fig.3**.

Acetyl-CoA acetyltransferase (Nmar_0841-Nmar1631)

Nmar_0841 and *Nmar_1631* correspond to K00626, Acetyl-CoA acetyltransferase, (putative 3-HP/4-HB Cycle enzyme), and this KO is therefore represented in **Fig.3**.

Metagenomes-Assembled Genomes

To assemble genomes bins, IDBA-UD[3] with default parameters was used to first assemble the metagenomes (**Supplementary Note 13**). Each sponge species was processed separately and the three samples from the control site were assembled independently from the three samples at the seep site, resulting in four distinct assemblies. Metagenome-assembled genomes (MAGs) were binned from each assembly using the Metabat default settings "very sensitive" and "super specific" options[4]. Within each site and species, genome bins were dereplicated using dRep[5] to discard bins with sequence similarity > 99%. Bin taxonomy was determined with GTDB-tk[6]. Archaeal bins from *C. singaporensis* at the control site were identical to archaeal bins from the seep site (metabatSS_coeloUpaCtrlOnly.26). Annotation was restricted to bins with a quality score (defined as completion – [5x contamination]) greater than 70% and was performed using EnrichM developed by J. Boyd (https://github.com/geronimp/enrichM). Only bins with quality scores >60% were submitted to NCBI (**Supplementary Table 20**).

Supplementary Notes

Supplementary Note 1: Taxonomic Assignment

The microbiome of each sponge species was taxonomically unique and sponge-associated communities were clearly distinct from the microbial community inhabiting the surrounding seawater (**Supplementary Figure 1**). In particular, Archaeal orders with relative abundance > 1% were only detected in sponge samples. A significant shift in the composition of the *C. singaporensis* microbiome was detected between sites **(Supplementary Figure 2)**, involving reduced relative abundances of Acidobacteria (from 4.1% to 1.2%), PAUC34f (from 5.3% to 0.4%) and Poribacteria (from 3.8% to 0.6%) at the seep. At the class level, differences included a reduction in Anaerolinea (from 3.5% to 1%) and Gammaproteobacteria (from 11.7% to 6%) at the seep. Within the Proteobacteria, the Rhodospirillales (class Alphaproteobacteria) were more abundant at the control than at the seep (4.6% versus 1.6%). Conversely, the genus Bdellovibrio (class Deltaproteobacteria) was more abundant at the seep (3% versus 0.%) as was the order EC94 (class Betaproteobacteria, 5.8% versus 0.2%). The *S. flabelliformis* microbiome showed fewer changes in community composition between sites, although a decline in the relative abundance of the order Thiohalorhabdales (from 26.6% to 13.9%) and the family Marinicellaceae (from 2.9% to 1.3%) was found at the seep.

Supplementary Note 2: Sample partitioning at the functional level

Similarity dendograms **(Supplementary Figure 3a)** and ordination analyses **(Supplementary Figure 3b** and **Supplementary Figure 4a)** of KO data revealed clear partitioning of the samples functions according to species and to a lesser extent, sites. Consistent with previous studies, seawater microbiomes were functionally distinct from the sponge-associated microbial communities.

Supplementary Note 3: General features of microbiomes

Microbiomes of the three species showed distinct features, including the number and proportion of significantly different genes enriched at the control or seep site (see main text and **Supplementary Table 1**).

Supplementary Note 4: Microbial functions in relation to environmental variables

Individual t-tests conducted on twenty-one environmental variables revealed that site had a significant effect on $p\mathrm{CO_2}$, pH, concentrations of CO_2, CO_3^{2-}, HCO_3^-, DIC, TA, and salinity (**Supplementary Tables 3-6**). However, the variation in salinity between sites was marginal (35 ± 0.63 ppt on average at the control and 34.8 ± 0.54 ppt on average at the seep, **Supplementary Table 3**). CAP analysis further revealed that the variables most strongly correlated to the distribution of KOs in the ordination were CO_2 concentration, $p\mathrm{CO_2}$, and CO_3^{2-} concentration (**Supplementary Figure 4** and **Supplementary Table 6**). Together these results support the primary role of inorganic carbon chemistry in driving the differences in microbial functions between the control and seep sites.

Supplementary Note 5: ABC transporters

In addition to the changes in relative abundance of ABC transporters presented in the main text, the microbiome of both *C. singaporensis* and *S. flabelliformis* at the seep exhibited a reduced potential for the uptake of oligopeptides (*oppABCDF*, 1.6-times in *C. singaporensis*, 2.3-times in *S. flabelliformis*), the antimicrobial compound microcin (*yejABEF*, 1.9-times in *C. singaporensis*, 1.7-times in *S. flabelliformis*) and iron (via the siderophore uptake system *ABC.FEV.APS* in *C. singaporensis*, 1.6–

times, and iron III uptake complex *afuABC* in *S. flabelliformis*, 1.5-times) (**Supplementary Figure 5** and **Supplementary Figure 6**). Furthermore, the microbiome of *C. singaporensis* at the seep had a significantly lower relative abundance of genes encoding sodium excretion systems (*natAB*, 11.8-times), uptake systems for molybdate and sulfur (*modABC*, 3-times), as well as an enhanced potential for uptake of the osmoprotectant sucrose (alpha-glucoside, *aglEFGK*, 4.3-times) and for release of inner membrane-bound lipoproteins into the periplasm, via the *lolCDE* complex (1.3-times). In contrast, the microbiome of *S. flabelliformis* at the seep exhibited a reduced potential for uptake of the osmoprotectant proline (*proVWX*, 1.6-times), perhaps compensated for by an increased relative abundance of the gene encoding transport/synthesis of the osmoprotectant glycine betaine (*opuABCD*, 3.2-times). P-value for genes encoding ABC transporters can be found in the Source data file.

Supplementary Note 6: Eukaryotic-like proteins

A few genes considered markers of host predation evasion were found in greater proportions at the CO_2 seep, with an increased relative abundance of tetratricopeptide repeats in the microbiomes of both species (5.4-times in *C. singaporensis*, 1.5-times in *S. flabelliformis*) (**Supplementary Figure 7**). Although these protein domains are characteristic of eukaryotic-like proteins (ELPs), which some sponge microbes express and likely use to escape host phagocytosis[7,8], it is difficult to confirm the biological meaning of these observations without additional analyses. Similarly, *nodI* and *nodJ*, two genes involved in the formation of bacterial nodules in legume species, had a higher relative abundance at the seep in the microbiomes of both species (3.7-times in *C. singaporensis*, 2.7-times in *S. flabelliformis*) (**Figure2**). In plant symbionts, these two genes encode proteins that export lipochitooligosaccharides or nodulation (nod) factors responsible for the establishment of symbiosis[9], although interpretation of our results is hindered by a lack of information on their role in sponges.

Supplementary Note 7: Carbonic anhydrase

Two genes encoding carbonic anhydrase were found in significantly lower relative abundance at the seep than at the control site in *C. singaporensis* microbiomes, which could indicate a diminished selection pressure at the seep on microbes which lack carbonic anhydrase (main text and **Supplementary Figure 9**).

Supplementary Note 8: Nitrogen cycle and glutamate-derived arginine biosynthesis in Archaea

The *C. singaporensis* microbiome exhibited a higher capacity for ammonium production via creatinine degradation at the control, and via creatine and arginine degradation at the seep (main text, **Supplementary Figure 10** and **Supplementary Table 8**). Arginine biosynthesis from glutamate can be divided into two phases: the first converting glutamate into ornithine, the second converting ornithine into arginine (**Supplementary Figure 11)**. In Bacteria such as *E. coli,* the first phase typically occurs via i) enzymes converting L-glutamate into N-acetylglutamate, encoded by *argA/J* and ii) a series of reactions converting N-acetylglutamate into ornithine, encoded by the *argBCDE* cluster (EC 2.3.1.1, 2.7.2.8, 1.2.1.38 and 2.6.1.11 successively) (**Supplementary Figure 11)**. The *argAJBCDE* cluster was found in the *C. singaporensis* metagenomes and, as expected, was almost exclusively assigned to bacterial scaffolds (**Supplementary Table 9)**. All genes showed a higher relative abundance at the control than at the seep, indicating a diminished capacity for bacteria to produce arginine at the seep

(**Supplementary Figure 12**). In Archaea, glutamate-driven arginine biosynthesis has only recently been characterised in *Sulfolobus acidocalcadarius*[10,11]. Arginine biosynthesis in Archaea follows a complex process involving an amino acid carrier protein which binds L-glutamate, encoded by the *lysW* gene. The first reaction corresponding to that catalysed by argA/J is performed by LysX-argX in the presence of the carrier protein LysW. The subsequent reaction catalysed by the LysZYJK complex leads to the biosynthesis of arginine or lysine, depending on whether the LyzX/argX initial substrate is α-aminoadipate (AAA) or L-glutamate, respectively. Substrate specificity is determined by the identity of residues 259 and 260 on the *lysX/argX* gene, with glycine-phenylalanine (Gly-Phe) leading to lysine biosynthesis and asparagine-serine (Asn-Ser) or asparagine-threonine (Asn-Thr) leading to arginine biosynthesis. All six genes (*lysW*, *lysX-argX*, *lysZ*, *lysY*, *lysJ* and *lysK*) were found in the metagenomes of *C. singaporensis* and all were more abundant at the seep than at the control site (**Supplementary Figure 12**). Inspection of residues 259 and 260 of the *lysX-argX* gene (K05827 in the KEGG Orthology nomenclature) confirmed the presence of both homologs (specifically glycine-lysine (Gly-Lys) and Asn-Thr at positions 259-260) and therefore the capacity to biosynthesize both lysine and arginine via this pathway (**Supplementary Table 10**). Whilst two-thirds of the *lysWXZYJK* gene cluster counts were found on archaeal scaffolds at the control, this proportion increased to 97% at the seep, suggesting that the increase in Archaea at the seep led to a rise in glutamate-driven arginine biosynthesis (**Supplementary Table 9)**. In contrast to *C. singaporensis*, *S. flabelliformis* microbiome displayed a general decreased potential for nitrogen cycling at the seep and consequently glutamine and glutamate biosynthesis (main text and **Supplementary Figure 13**) with almost no contribution from Archaea (**Supplementary Table 11**).

Supplementary Note 9: Taurine and homoserine degradation

As reported in the main text, the genomic potential for taurine degradation and associated sulfur assimilation were reduced at the seep in *S. flabelliformis*. This was supported by the relative abundances of seven genes involved in these pathways (**Supplementary Figure 14** and **Supplementary Table 12**).

Supplementary Note 10: Sulfur metabolism

C. singaporensis microbiomes presented a reduced potential for sulfur assimilation at the seep, indicated by the 10-fold significant decrease in relative abundance of both subunits of the adenylylsufate reductase (*aprA* and *aprB*, 11.5-times and 9.8-times respectively, ANOVA $p < 0.01$ for both), which catalyses the interconversion of adenylylsulphate into sulfite and vice versa (**Supplementary Figure 15**). Both subunits of the dissimilatory sulfite reductase (dsrA and dsrB) also showed a large decrease at the seep (5.7-times and 6.2-times, ANOVA $p < 0.01$ for both). Lower proportions of many taxa could have led to this decreased potential for sulfate reduction at the seep (**Supplementary Table 13**). In contrast, the *S. flabelliformis* microbiome might benefit from a greater potential for energy production via thiosulfate oxidation at the seep, with thiosulfate potentially originating from the seep environment. This enhanced capacity could arise from a (mostly non-significant) increase in the relative abundance of four of the seven genes encoding the *sox* cluster *soxXYZABCD*, namely *soxC*, *soxZ*, *soxY*, *soxX* (4.2-times, 1.9-times, 2.4-times, 2.4-times respectively) (**Supplementary Figure 16**). The *sox* complex genes were mostly assigned to *Gammaproteobacteria* (**Supplementary Table 14**), including purple-sulfur bacteria (*Chromatiales*), perhaps indicating that the thiosulfate could be used for anoxygenic photosynthesis.

Supplementary Note 11: Cobalamin biosynthesis

Microbial symbionts of *C. singaporensis* possessed the complete pathway for cobalamin (vitamin B12) biosynthesis and the relative abundance of these genes was 60% higher in sponges at the CO_2 seep (totalling 1% vs 0.6% average relative abundance at the seep and control, respectively (ANOVA, p = 0.03); **Figure2b**, **Figure2c** and **Supplementary Figure 17**). Cobalamin biosynthesis, a major component of the porphyrin and chlorophyll metabolism KEGG pathway, can take place both aerobically and anaerobically, however oxygen availability did not have a significant effect on cobalamin biosynthesis genes (**Supplementary Table 15**). Scaffolds carrying cobalamin synthesis genes from both the aerobic and anaerobic pathways primarily originated from Thaumarchaeota (26% at the seep vs 19% at the control), followed by Proteobacteria and Cyanobacteria (**Supplementary Table 16**). While this suggests that sponge-associated Thaumarchaeota can use aerobic and anaerobic pathways as has been reported for free-living marine Thaumarchaeota[12], "anaerobic" vitamin B12 biosynthesis would actually happen under low-oxygen, rather than truly anoxic conditions, given that Thaumarchaeota require O_2 to perform ammonia oxidation[12,13]. Genes encoding three cobalamin-dependent enzymes were detected in the *C. singaporensis* microbiome, with no significant difference in relative abundance between sites (data not shown). The higher microbial potential for cobalamin biosynthesis at the seep without a broadly higher potential for its utilisation suggests that cobalamin might be predominantly used by the sponge host. Differential utilisation of cobalamin amongst microbial taxa was evident across sites though, with the cobalamin-dependent enzyme methylmalonyl-coA mutase (part of the 3-Hydropropionate/4-hydroxybutyrate (HP/HB) cycle) largely present on bacterial or unclassified scaffolds at the control site (26% ± 17%) but on Thaumarchaeota scaffolds at the seep (81% ± 4%).

Supplementary Note 12: Thiamine biosynthesis

An increased capacity for thiamin (vitamin B1) biosynthesis was evident in the *S. flabelliformis* microbiome at the seep. Vitamin B1 is synthesized *de novo* by 76% of cultured marine bacteria[14]. Thiamin biosynthesis takes place in four phases: i) synthesis of 4-amino-5-hydroxymethylpyrimidine diphosphate (HMP moiety), ii) synthesis of 4-methyl-5-(2-phosphoethyl)-thiazole (THZ-P moiety), iii) linking these two compounds to form thiamin monophosphate (TMP) and iv) phosphorylation of TMP into thiamin pyrophosphate (TDP), the active form of vitamin B1[15]. Some microbial species have the capacity to synthesize only either HMP or THZ-P, whilst acquiring the missing compound from the environment. *S. flabelliformis* symbionts had the potential to synthesize HMP *de novo* via the degradation of 5-amino-1-(5-phospho-β-D-ribosyl) imidazole (AIR), a product of purine metabolism. The two genes required, *thiC* and *thiD*, were significantly more abundant (2-3 times) at the seep than at the control site (**Supplementary Figure 18**). All genes required for the synthesis of THZ-P from 2-glyceraldehyde-3-phosphate, glycine and cysteine were present in the metagenome, with the exception of *thiF*. The two genes involved in glycine degradation, *thiO* and *thiG*, were also significantly more abundant (2-3 times) at the seep, as were *thiE* and *thiL* which encode the last two enzymes for the successive formation of TMP and TDP. Scaffolds carrying these genes were primarily assigned to Proteobacteria, Thaumarchaeota and unclassified phyla (**Supplementary Table 17**). In addition, Cyanobacteria contributed a notable proportion of scaffolds carrying *thiO* and *thiG*. No consistent pattern was found in the taxonomic distribution of scaffolds that could explain the significant increase in the relative abundance of *thiO*, *thiG*, *thiC, thiD, thiE* and *thiL* at the seep.

Supplementary Note 13: Metagenome-assembled genomes

Supplementary Table 20 provides details of the Metagenome-assembled Genomes. All KOs listed as thaumarchaeal for the HP/HB cycle in **Supplementary Table 7,** and for urease, agmatinase and arginine decarboxylase in **Supplementary Table 8** were found in the *Nitrosopumilus* bin metabatVS_coeloUpaCtrlOnly.21 (**Supplementary Table 20**), confirming that *C. singaporensis* possesses *Nitrosopumilus* symbionts with the genomic potential to perform the HPHB cycle described in **Figure3a** and urease formation and degradation described in **Figure3b**.

Supplementary references

1 Konneke, M. *et al.* Ammonia-oxidizing archaea use the most energy-efficient aerobic pathway for CO2 fixation. *Proc. Natl. Acad. Sci. U. S. A.* **111**, 8239-8244 (2014).

2 Qin, W. *et al.* Stress response of a marine ammonia-oxidizing archaeon informs physiological status of environmental populations. *ISME J.* **12**, 508-519 (2018).

3 Peng, Y., Leung, H. C. M., Yiu, S. M. & Chin, F. Y. L. IDBA-UD: a de novo assembler for single-cell and metagenomic sequencing data with highly uneven depth. *Bioinformatics* **28**, 1420-1428 (2012).

4 Kang, D. D., Froula, J., Egan, R. & Wang, Z. MetaBAT, an efficient tool for accurately reconstructing single genomes from complex microbial communities. *PeerJ* **3**, e1165 (2015).

5 Olm, M. R., Brown, C. T., Brooks, B. & Banfield, J. F. dRep: a tool for fast and accurate genomic comparisons that enables improved genome recovery from metagenomes through de-replication. *ISME J.* **11**, 2864 (2017).

6 Parks, D. H. *et al.* A standardized bacterial taxonomy based on genome phylogeny substantially revises the tree of life. *Nat. Biotechnol.* **36**, 996 (2018).

7 Reynolds, D. & Thomas, T. Evolution and function of eukaryotic-like proteins from sponge symbionts. *Mol. Ecol.* **25**, 5242-5253 (2016).

8 Diez-Vives, C., Moitinho-Silva, L., Nielsen, S., Reynolds, D. & Thomas, T. Expression of eukaryotic-like protein in the microbiome of sponges. *Mol. Ecol.* **26**, 1432-1451 (2017).

9 Cardenas, L., Dominguez, J., Santana, O. & Quinto, C. The role of the nodI and nodJ genes in the transport of Nod metabolites in Rhizobium etli. *Gene* **173**, 183-187 (1996).

10 Ouchi, T. *et al.* Lysine and arginine biosyntheses mediated by a common carrier protein in Sulfolobus. *Nat. Chem. Biol.* **9**, 277 (2013).

11 Tomita, T. Structure, function, and regulation of enzymes involved in amino acid metabolism of bacteria and archaea. *Biosci. Biotechnol. Biochem.* **81**, 2050-2061 (2017).

12 Doxey, A. C., Kurtz, D. A., Lynch, M. D. J., Sauder, L. A. & Neufeld, J. D. Aquatic metagenomes implicate Thaumarchaeota in global cobalamin production. *ISME J.* **9**, 461-471 (2015).

13 Peng, X. F., Jayakumar, A. & Ward, B. B. Community composition of ammonia-oxidizing archaea from surface and anoxic depths of oceanic oxygen minimum zones. *Front. Microbiol.* **4** (2013).

14 Sañudo-Wilhelmy, S. A., Gómez-Consarnau, L., Suffridge, C. & Webb, E. A. The Role of B Vitamins in Marine Biogeochemistry. *Annu. Rev. Mar. Sci.* **6**, 339-367 (2014).

15 Park, J.-H. *et al.* Biosynthesis of the Thiazole Moiety of Thiamin Pyrophosphate (Vitamin B1). *Biochemistry* **42**, 12430-12438 (2003).
