# Supplementary material for: Changes in the metabolic potential of the sponge microbiome under ocean acidification
Source: Nat Commun. 2019 Sep 12;10:4134. doi: 10.1038/s41467-019-12156-y (PMC6742649; doi:10.1038/s41467-019-12156-y)
Supplement: Supplementary file 2 — Reporting Summary [file 41467_2019_12156_MOESM2_ESM.pdf]

nature research

Corresponding author(s): NICOLE S. WEBSTER

Last updated by author(s): Jul 30, 2019

Reporting Summary

Nature Research wishes to improve the reproducibility of the work that we publish. This form provides structure for consistency and transparency in reporting. For further information on Nature Research policies, see Authors & Referees and the Editorial Policy Checklist.

Statistics

For all statistical analyses, confirm that the following items are present in the figure legend, table legend, main text, or Methods section.

n/a	Confirmed	
☐	☒	The exact sample size (n) for each experimental group/condition, given as a discrete number and unit of measurement
☐	☒	A statement on whether measurements were taken from distinct samples or whether the same sample was measured repeatedly
☐	☒	The statistical test(s) used AND whether they are one- or two-sided<br>*Only common tests should be described solely by name; describe more complex techniques in the Methods section.*
☐	☒	A description of all covariates tested
☐	☒	A description of any assumptions or corrections, such as tests of normality and adjustment for multiple comparisons
☐	☒	A full description of the statistical parameters including central tendency (e.g. means) or other basic estimates (e.g. regression coefficient) AND variation (e.g. standard deviation) or associated estimates of uncertainty (e.g. confidence intervals)
☐	☒	For null hypothesis testing, the test statistic (e.g. F, t, r) with confidence intervals, effect sizes, degrees of freedom and P value noted<br>*Give P values as exact values whenever suitable.*
☒	☐	For Bayesian analysis, information on the choice of priors and Markov chain Monte Carlo settings
☒	☐	For hierarchical and complex designs, identification of the appropriate level for tests and full reporting of outcomes
☒	☐	Estimates of effect sizes (e.g. Cohen's d, Pearson's r), indicating how they were calculated

Our web collection on statistics for biologists contains articles on many of the points above.

Software and code

Policy information about availability of computer code

Data collection	Data collection was performed using software IDBA-UD/1.1.1 , bowtie/2.2.4, samtools/1.2 , python/2.7.9 and python/2.7.12, prodigal/2.6.1, hmmer/3.1b2, pplacer/1.1.alpha16, metabat/0.2.4.
Data analysis	Data analysis was performed using R/3.4.1 and R packages Vegan, CAR, MatrixStats, tidyr, dplyr, tibble, ggplot2, as well as PRIMER/ PERMANOVA V6 and V7.

For manuscripts utilizing custom algorithms or software that are central to the research but not yet described in published literature, software must be made available to editors/reviewers. We strongly encourage code deposition in a community repository (e.g. GitHub). See the Nature Research guidelines for submitting code & software for further information.

Data

Policy information about availability of data

All manuscripts must include a data availability statement. This statement should provide the following information, where applicable:

- Accession codes, unique identifiers, or web links for publicly available datasets
- A list of figures that have associated raw data
- A description of any restrictions on data availability

Raw reads and Metagenomes-Assembled Genomes are available from the NCBI SRA under accession number SRP159543 [https://www.ncbi.nlm.nih.gov/sra/?term=SRP159543]. Assemblies (see Supplementary Table 18 for assemblies ID) are available from IMG-MER [https://img.jgi.doe.gov/cgi-bin/m/main.cgi]. The source data underlying all figures and Supplementary Figures, except Supplementary Fig. 11 are provided as two Source Data files, one for each sponge species

Field-specific reporting

Please select the one below that is the best fit for your research. If you are not sure, read the appropriate sections before making your selection.

☐ Life sciences ☐ Behavioural & social sciences ☒ Ecological, evolutionary & environmental sciences

For a reference copy of the document with all sections, see nature.com/documents/nr-reporting-summary-flat.pdf

Ecological, evolutionary & environmental sciences study design

All studies must disclose on these points even when the disclosure is negative.

Study description	This study investigated the changes occurring in the functional potential of the microbiome of two marine sponge species living in low pH, "acidified" waters and adjacent (~ 500m away) ,"control" waters, at shallow coral reefs CO2 seep sites in Papua New Guinea.
Research sample	Three samples of each of two sponge species were collected. The species were Coelocarteria singaporensis and Stylissa flabelliformis. These particular species were chosen based on a previous study (Morrow et al., 2015) characterizeing the microbial communities of the 3 species at these particular field sites.
Sampling strategy	Sample size of three samples per species was chosen based on the following criteria: 1) minimizing impact on the field site, 2) accounting for statistical testing sample size requirements, 3) space availability for storage in the field and during transport, 4) cost (temporal and financial) of sample processing and sequencing.
Data collection	Data collection was performed by E.S.B. Environmental data were collected over multiple expeditions as well as via remote loggers. Environmental data collection times collection dates are available from Supplementary Tables 3, 4 and5.
Timing and spatial scale	Samples were collected between the 25th and the 30th of March 2014 during daytime. All samples could not be collected on the same day due to time restrictions inherent to SCUBA diving.
Data exclusions	No data was excluded.
Reproducibility	Non-applicable due to the field-based nature of the study.
Randomization	Non-applicable due to the field-based nature of the study.
Blinding	Blinding was not applied to the data as it did not fit the type of analyses carried out.

Did the study involve field work? ☒ Yes ☐ No

Field work, collection and transport

Field conditions	Our manuscript contains analysis of more than 20 environmental variables collected in the field. Details are given in Supplementary Tables 3, 4, 5 and 6.
Location	Field work was carried out in the Milne Bay Province in the d'Entrecasteaux Channel in Papua New Guinea at a depth of 5m. Control site GPS coordinates: 9.828217 S 150.820517 E, Seep site GPS coordinates: 9.8241 S 150.825833 E.
Access and import/export	Australian permit (#14002493) was obtained for the import of marine sponges from Papua New Guinea. Local populations were informed of the sampling expedition on the reef ahead of arrival and contact was established with the local authority on arrival, prior to any scientific activity, as traditionally required in Papua New Guinea.
Disturbance	As any human activity, field collection could have disturbed habitat; however all precautions were taken to minimize such disturbances, such as periods of rest between dives, collections restricted to day time and good buoyancy control during diving.

Reporting for specific materials, systems and methods

We require information from authors about some types of materials, experimental systems and methods used in many studies. Here, indicate whether each material, system or method listed is relevant to your study. If you are not sure if a list item applies to your research, read the appropriate section before selecting a response.

Materials & experimental systems

n/a	Involved in the study	
☒	☐	Antibodies
☒	☐	Eukaryotic cell lines
☒	☐	Palaeontology
☐	☒	Animals and other organisms
☒	☐	Human research participants
☒	☐	Clinical data

Methods

n/a	Involved in the study	
☒	☐	ChIP-seq
☒	☐	Flow cytometry
☒	☐	MRI-based neuroimaging

Animals and other organisms

Policy information about studies involving animals; ARRIVE guidelines recommended for reporting animal research

Laboratory animals	Non-applicable
Wild animals	Non-applicable
Field-collected samples	Samples of marine sponges were collected on SCUBA into individual plastic bags and immediately snap-frozen on the research vessel.
Ethics oversight	Ethics approval is not required for marine sponge sampling.

Note that full information on the approval of the study protocol must also be provided in the manuscript.
